# Effect of Hybrid Fiber Compositions on Mechanical Properties and Durability of Ultra-High-Performance Concrete: A Comprehensive Review

**DOI:** 10.3390/ma18112426

**Published:** 2025-05-22

**Authors:** Paulina Dziomdziora, Piotr Smarzewski

**Affiliations:** 1 Doctoral School, Faculty of Civil Engineering and Geodesy, Military University of Technology, 2 Gen. Sylwestra Kaliskiego, 00-908 Warsaw, Poland; paulina.dziomdziora@student.wat.edu.pl; 2 Faculty of Civil Engineering and Geodesy, Military University of Technology, 2 Gen. Sylwestra Kaliskiego, 00-908 Warsaw, Poland

**Keywords:** ultra-high-performance concrete, hybrid fiber, mechanical properties, durability

## Abstract

Ultra-high-performance concrete (UHPC) has emerged as a revolutionary material in structural engineering due to its exceptional mechanical properties and durability. This review comprehensively examines the influence of hybrid fiber compositions on UHPC, focusing on mechanical performance and resistance to environmental degradation. Hybrid fibers, which combine steel and synthetic and basalt fibers, improve compressive, tensile, and flexural strengths by bridging microcracks and limiting macrocrack propagation. Studies reveal that steel fiber combinations, particularly those with varying lengths and shapes, significantly improve ductility and load-bearing capacity, while steel–synthetic hybrids balance strength and flexibility. However, excessive synthetic fibers can reduce compressive strength. Basalt–synthetic hybrids, though less effective in compression, excel in tensile strength and crack resistance. Durability assessments highlight the superior resistance of UHPCs to chloride penetration, carbonation, freeze–thaw cycles, and high temperatures, and hybrid fibers further mitigate spalling and permeability. Polypropylene fibers, for instance, enhance fire resistance by creating vapor release channels. The challenge of optimizing fiber proportions and mix designs remains to minimize trade-offs between strength and workability. Future research should explore advanced fiber combinations, long-term environmental performance, and eco-friendly additives to expand the applicability of UHPC in sustainable infrastructure. This review underscores the potential of hybrid fibers to tailor UHPCs for diverse engineering demands while addressing current limitations.

## 1. Introduction

Conventional concrete (OC) is widely used in structural engineering because of its access to construction and maintenance costs, as well as its durability and construction possibilities. However, its low durability makes it susceptible to cracking and complete failure of load-bearing capacity after damage, coexisting with an impact on its performance parameters [1,2]. The answer to the limitations of traditional concrete is high-performance concrete (HPC). It is characterized by greater strength, better durability, and resistance to external factors. It is distinguished by a fast hardening rate, high tightness, and low permeability, which makes it resistant to water, de-icing salts, and other aggressive factors [3,4,5,6]. Due to these properties, it is widely used in structures with large spans and heights [7,8]. Ultra-high-performance concrete (UHPC) is an advanced construction material that is gaining increasing recognition in structural engineering due to its exceptional mechanical and durability properties. It is considered a future solution to sustainable infrastructure. This material, developed since the 1990s, is characterized by an extraordinary compressive strength of up to 300 MPa, as well as an exceptional tensile strength, which usually exceeds 5 MPa [9,10,11,12,13,14,15,16]. UHPC is distinguished by its ability to maintain structural properties even after fracture when fibers are used, which resembles the behavior of metals rather than traditional, brittle concretes [12,17,18,19]. The main feature that distinguishes UHPC from other types of concrete is its optimized microstructure, which is achieved by a low water-to-binder ratio (w/b), the use of fine aggregate, and a high binder concentration. Due to these properties, the porosity of concrete is significantly reduced, which translates into its excellent durability and resistance to various external factors. A key role in obtaining these parameters is also played by using a superplasticizer (SP), which ensures the high fluidity of the mixture, allowing its homogeneous consolidation and limiting the porosity of the composite [9,12,20,21,22,23,24,25]. The UHPC is also distinguished by its exceptional durability under difficult environmental conditions. Thanks to its compact microstructure and resistance to atmospheric, chemical, and mechanical factors, this concrete is used in the construction of structures exposed to extreme loads, such as bridges, wind towers, marine structures, and hydrotechnical structures [4,5,21,26,27,28]. Due to the use of UHPC fibers, it is also characterized by high ductility and resistance to crack propagation, making it similar to metallic materials in this respect [9,12,13,14,15,16,20,21,22,23,24,29].

High-performance and ultra-high-performance concretes are widely used in construction because of their exceptional mechanical properties and durability. The high strength and resistance to external factors make these materials particularly useful in structures that require high load-bearing capacity, long-lasting life, and high resistance to mechanical and chemical damage. UHPCs are used primarily in infrastructure construction, where maximum durability and minimal maintenance costs are the focus over a long period of use. It is used in the construction of bridges, including large spans and road infrastructure elements, such as bridge deck overlays or prefabricated connection elements. High tensile and compressive strength allows the use of UHPC in structures with very high dynamic loads, e.g., foundations of high-rise buildings [9,30,31,32]. UHPCs are also widely used in the modernization and repair of existing structures. Due to their unique ability to limit crack propagation and high impermeability, they are perfect as a repair material for the renovation of damaged structural elements, extending their service life and improving the safety of use [33,34,35]. High-performance and ultra-high-performance concretes are currently used around the world, including Australia, Austria, Canada, China, France, Germany, Malaysia, Spain, Italy, Croatia, Japan, South Korea, Switzerland, and the United States. Most of the implementation projects were inspired by government entities as pilot investments to stimulate future larger-scale applications of UHPC [26,27,33].

Despite its numerous advantages, UHPC also has some disadvantages that limit its widespread use. The main problem is the high cost of production, related to the use of a large amount of cement (950 to 1000 kg/m^3^) and silica dust (100 to 250 kg/m^3^), which results in increased emissions of CO_2_ [25,36,37,38]. Furthermore, this concrete, without fibers, is characterized by high brittleness, and its modulus of elasticity is 45–60 GPa, which is an undesirable feature in some applications [4,39,40,41,42]. It should be noted that the very tight structure of UHPC can lead to explosive spalling under fire conditions. The high density and low water permeability of this material mean that internal water can cause rapid cracking and concrete damage due to elevated temperatures, which is a significant problem in applications exposed to fire.

The introduction of fibers into concrete mixtures significantly affects their mechanical and functional properties, changing the characteristics of the material and expanding the range of its potential applications. Fibers act as reinforcement elements that delay crack formation and propagation, directly affecting the increase in tensile strength, plasticity, and energy absorption capacity of concrete [43,44,45]. Due to this, the UHPC composite changes its properties from ceramic brittleness to metallic plasticity, which is a significant advantage in the context of structural applications [46,47,48]. To further optimize the mechanical properties of UHPC and maintain the desired workability, attempts are being made to hybridize fibers, i.e., to combine different types of fibers in one blend.

In this article, the concept of hybrid fibers refers to the intentional combination of fibers with different physical and mechanical characteristics, such as length, modulus of elasticity, tensile strength, and ductility. This approach aims to achieve a synergistic effect that enhances both the properties of fresh and hardened concrete. The idea of ‘hybrid fiber reinforcement’ has been present in engineering practice since at least the early 2000s. One of the first significant works that introduced this terminology was the publication by Banthia and Gupta [49], where the authors described ‘Hybrid Fiber Reinforced Concrete (HyFRC)’ as a system that combines various types of fibers to improve the performance of concrete in a comprehensive and synergistic way.

Hybridization involves mixing fibers of different geometry, length, and material. For example, combining longer and shorter steel fibers in the right proportions can significantly improve bending strength, as noted in numerous studies [50,51,52,53,54,55]. The addition of fibers also affects other properties of concrete. Fibers reduce the formation of shrinkage cracks and reduce the brittleness of the material. In addition, they improve its durability by strengthening the microstructure of the concrete and limiting the propagation of damage. The variety of possible combinations of fibers provides a wide range of possibilities in the design of UHPCs with custom properties depending on the structural requirements. However, more research is needed on the synergistic effect of different types of fibers in hybrid mixtures, as well as on the effect of fiber length, shape, and proportion on the final properties of concrete.

The influence of fibers on the mechanical and durability properties of UHPC will be discussed in more detail later in this article, taking into account the results of previous research and new directions of development in this field.

## 2. Methodology of Literature Review

To prepare the review article, a systematic literature search was conducted in reputable databases such as ScienceDirect and Scopus. The selection process included the analysis of titles, keywords, and abstracts, focusing on articles related to the use of different fiber mixtures in UHPC. Special attention was paid to publications that contained experimental results of mechanical and durability tests of hybrid fiber concrete. The time period of the literature search for durability studies of UHPC with the addition of hybrid fibers covered the years 2003–2024, while for mechanical studies of UHPC with hybrid fibers, it covered the years 2012–2024. Table 1 presents key information on the reviewed studies, and Table 2 presents a summary of the most important information on the durability studies reviewed.

The review selection process was based on the analysis of parameters, such as the type and volume fraction of fibers, fiber shape, their orientation, casting method, and the influence of these factors on the mechanical properties of concrete. In addition to thematic relevance, more detailed inclusion criteria were applied to ensure the reliability of the analyzed data. Specifically, (i) the studies had to report experimental findings rather than solely numerical or theoretical analyses; (ii) the volume fraction of fibers had to be clearly specified; (iii) the type and characteristics of the fibers (e.g., material, shape, size) had to be explicitly described; and (iv) the results needed to include an assessment of at least one mechanical or durability-related property.

To guarantee the high quality of the selected publications, each study was critically evaluated with respect to (i) the clarity and completeness of the experimental procedures; (ii) the adequacy and standardization of the test methods used (e.g., compliance with recognized standards); (iii) the reproducibility and statistical significance of the results; and (iv) the extent to which the interactions between fibers and the UHPC matrix were discussed, as well as the overall quality of the journals in which the studies were published. Studies with unclear methodologies or incomplete results were excluded from the detailed analysis. The selected publications were further evaluated on the basis of experimental methods performed, such as tensile, bending, and compression tests. The aim was to provide a comprehensive and reliable review of the current state of knowledge.

In the course of our review, we considered performing a meta-analysis; however, several challenges prevented this. These included significant heterogeneity in experimental methods, high variability in fiber types, doses, and hybridization strategies, and inconsistent reporting of key statistical data, such as standard deviations or sample sizes. Additionally, the studies focused on different performance aspects, such as compressive or flexural strength, toughness, or durability, often under varying conditions. Given these limitations, we determined that a qualitative synthesis would provide a more coherent and informative summary of the current research. Although there are reviews of fiber-reinforced cement composites, many of them mainly focus on cement composites or conventional concrete, with limited emphasis on hybrid fiber-reinforced ultra-high-performance concrete. Existing works often investigate selected aspects, such as tensile behavior, microstructure, or sustainability, but rarely offer a comprehensive integration of mechanical performance and durability, especially from an experimental perspective.

This article attempts to fill the gaps in the literature by comprehensively presenting the impact of hybrid fiber use on the mechanical properties and durability of UHPC, taking into account the current state of knowledge, technological challenges, and future directions of development. Particular attention is paid to the synergistic effect of fiber hybridization and its microstructural interactions, which are key to further improving UHPC and its wider application in sustainable construction.

## 3. Characteristics of Fiber

Fibers in concrete are small elements with specific physical and mechanical properties that are introduced into the concrete mix to improve its parameters. They can be made of various materials, such as steel, glass, polypropylene, basalt, or carbon. The role of fibers is to increase tensile strength, crack resistance, and improve other properties, such as durability, resistance to salts, or behavior under the influence of high temperatures. Fibers also act as dispersed reinforcement, limiting the development of microcracks and increasing the structural cohesion of concrete.

The history of fiber use in construction is an evolution from simple natural materials such as straw to technologically advanced synthetic and composite fibers that are resistant to extreme conditions. The introduction of each type of fiber was a response to specific needs and challenges in construction, allowing the creation of increasingly durable, flexible, and resistant structures. Modern fibers (glass, basalt, synthetic, and carbon) continue to play a key role in the development of building materials technology.

Fibers can be divided into categories based on their composition: non-synthetic fibers and synthetic fibers [12], on their size: microfibers (6–20 mm) that sew potential and initial cracks in concrete [80] and macrofibers (usually over 30 mm), which start to act after this initial phase and are responsible for plasticity and crack width reduction [81], and on their modulus of elasticity: low-modulus fibers (including polypropylene, polyethylene, and polyolefin) and high-modulus fibers (including steel, glass, carbon) [82], as well as their shape (e.g., straight, hooked, twisted) [82,83,84].

### 3.1. Non-Synthetic Fibers

Non-synthetic fibers include primarily steel, basalt, glass, and carbon fibers. Their common feature is their high mechanical strength, the ability to improve concrete properties, and a variety of shapes and sizes. They differ in corrosion resistance, environmental impact, and production costs [66,85,86].

#### 3.1.1. Steel Fibers

**Figure 1:**
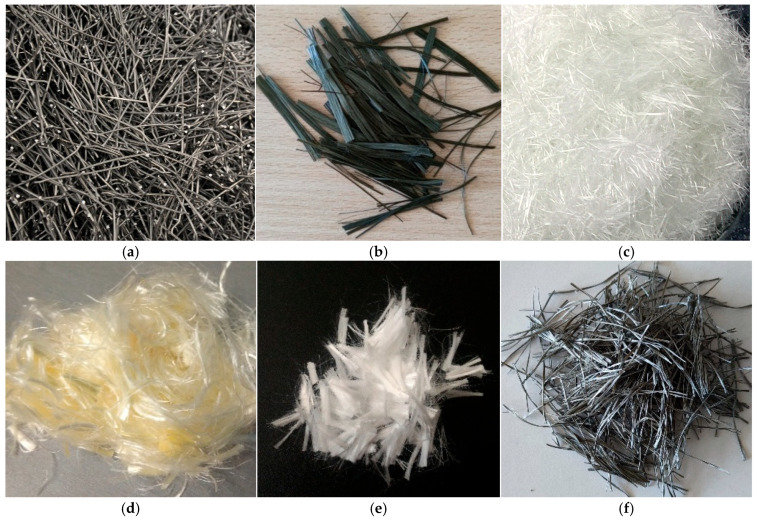
Examples of fiber types used by authors in hybrid fiber-reinforced UHPC/HPC: (**a**) steel hooked, (**b**) basalt straight, (**c**) glass straight, (**d**) polyvinyl alcohol straight, (**e**) polypropylene straight, (**f**) polyolefin straight.

Steel fibers (SFs) are the most commonly used type of reinforcement in UHPC [12]. They are characterized by high tensile strength, high modulus of elasticity, and the ability to improve the compressive, bending, and tensile strength of concrete, as well as the ability to absorb energy [19,65,82,87,88,89,90,91,92,93,94,95,96,97,98,99,100,101,102,103,104,105,106]. The variety of shapes and sizes of steel fibers affects their utility properties. In terms of length, these fibers are divided into ultra-short (L_f_ ≤ 8 mm), short (8 mm ≤ L_f_ ≤ 13 mm), long (13 mm ≤ L_f_ ≤ 30 mm), and ultra-long (L_f_ > 30 mm). In terms of diameter, microfibers (d_f_ ≤ 0.8 mm), fine (0.8 mm ≤ d_f_ ≤ 1 mm), and regular (d_f_ > 1 mm) are distinguished [21]. Depending on the geometry, steel fibers can take different forms, including corrugated (CSF), hooked (HSF) (see Figure 1a), and twisted (TSF) [107].

Despite their numerous advantages, steel fibers are also environmentally and energetically burdensome. Their production requires large amounts of natural raw materials and high temperatures, which are associated with high costs [108,109,110]. Furthermore, steel fibers are susceptible to corrosion, which can reduce their ability to form a permanent bond with the concrete matrix [12,111,112,113]. The high strength of steel fibers and their good adhesion to the binder make them popular in fiber-modified concretes, although their susceptibility to corrosion remains a significant challenge [85,86]. To increase corrosion resistance, stainless steel and protective coatings are used [114]. In traditional FRC, 0.25% to 2% of the volume of steel fiber is used, while in UHPC, this share can reach 6% [20,96].

#### 3.1.2. Basalt Fiber

Basalt fiber (BF) is a natural material consisting of silica, alumina, iron oxide, calcium oxide, and other components, formed from melted volcanic basalt rock at a temperature of 1500–1700 °C (see Figure 1b) [115,116]. They are characterized by high tensile strength (up to 4000 MPa), high modulus of elasticity, chemical resistance, and stability in alkaline and acidic environments [90,109,117,118,119,120]. Furthermore, they show significant corrosion resistance in alkaline environments and good stability in acidic environments [12,121,122,123]. The operating temperature range of basalt fibers is 200 to 800 °C, but at higher temperatures, changes in their structure may occur [124]. Their production is characterized by relatively low cost and less environmental burden than steel fibers. Due to these properties, basalt fibers are a promising alternative to UHPC, although research on their long-term impact on composites is still lacking [12].

#### 3.1.3. Glass Fiber

Glass fibers (GFs) offer high tensile strength at low weight (in Figure 1c) [80,125]. They have been used since 1931 as concrete reinforcement. Their main limitations are their brittleness and low resistance to alkali [126], making their long-term use in aggressive environments difficult. Additionally, their production is associated with a large carbon footprint and high chemical consumption [127].

#### 3.1.4. Carbon Fiber

Carbon fibers (CFs), consisting of long chains of carbon atoms, are characterized by exceptional tensile strength, low weight, high thermal and electrical conductivity, and chemical stability [128,129,130,131]. However, their production requires significant energy input and is associated with high greenhouse gas emissions [130,131]. In addition, it is extremely flexible and shows a high resistance to fatigue of the material during load and unload cycles [132,133]. Carbon fibers are classified by geometry into carbon nanofibers and uniform length fibers. Additionally, they can also be classified by size. Fibers with less than 24,000 tons are referred to as standard fibers, while those with a weight greater than 50,000 tons are classified as high-tooth fibers [134].

### 3.2. Synthetic Fibers

Synthetic fibers are widely used in construction, and their diversity is due to the wide range of plastics, such as polyvinyl alcohol (PVA) (Figure 1d), polypropylene (PP) (Figure 1e), polyolefin (Figure 1f), and polyethylene (PE) [135,136,137]. The polymers from which synthetic fibers are made consist of long repeating chains of molecules connected by intermolecular interactions [138]. Their properties are due to the level of order in their structure and can be classified as crystalline, semi-crystalline, or amorphous [139,140]. Polymer fibers can be derived from virgin and recycled raw materials, which is an important solution for waste reuse [138]. Their key advantage is corrosion resistance and low weight [141,142], although their adhesion to the cement matrix is limited due to their hydrophobic nature [143]. The production of synthetic fibers has some environmental impact, but it is less than that of steel fibers [143,144].

PP fibers are popular due to their low cost, high pH resistance, and easy dispersion [145]. The resistance of concrete to cracking is improved by reducing the width and number of shrinkage cracks by up to 99% [137,146]. However, they have a low modulus of elasticity and limited resistance to high temperatures [147,148,149,150].

PVA fibers are characterized by a high modulus of elasticity (29–42 GPa) and good adhesion to the cement matrix due to the presence of hydroxyl groups [151,152,153]. However, they are more expensive than other polymers and have limited resistance to cracking [154,155].

PE fibers have high tensile strength (up to 3.5 GPa) and modulus of elasticity of up to 110 GPa [156,157,158]. However, their hydrophobic nature makes it difficult to bond effectively with cement. In UHPC, PE fibers increase the ability to withstand tensile stress and absorb energy [89,116,159,160,161,162].

Synthetic fibers, despite certain limitations, play an important role in UHPC, helping to control microcracks and improving resistance to abrasion and high temperatures [21,109,117,157,159,161,163,164,165,166,167,168,169].

### 3.3. Influence of Fiber Orientation and Casting Methods on UHPC Properties

The orientation of fibers in the UHPC matrix has a significant impact on the mechanical properties of the material, such as tensile strength, bending strength, and ductility. Fibers arranged in the direction of stress more effectively bridge cracks, improve resistance to pulling out, and increase load transfer efficiency [12,25].

The tests carried out have shown that optimal properties are obtained when the fibers are oriented at an angle of 30 to 45° to the stretching direction [170,171]. In such a configuration, the highest pulling forces are obtained, and a significant increase in the number of fibers in the crack plane is achieved, which intensifies the bridging effect [172,173]. Controlled fiber orientation along the stretching direction can lead to a significant increase in the bending strength, tensile strength, and ductility of the material [174,175].

The literature also indicates the most effective practices supporting fiber orientation in UHPC. One of the most effective methods is to guide the casting from one end of the mold to the other. Due to the high viscosity of the fresh UHPC mixture and the presence of shear rate gradients, the fibers tend to align along the flow direction. Studies have shown that this approach leads to better fiber orientation and, therefore, higher tensile strength—up to 20% compared to central casting [174,176,177]. Furthermore, the use of inclined chutes or extrusion nozzles can increase tensile strength by up to 109% [177]. Another approach is to use the magnetization of steel fibers. By giving the fibers the same magnetic polarity, they are mutually repelled and aligned along the magnetic field lines. This method allows an increase in tensile strength of 16–50% [178,179,180,181]. In turn, mechanical devices, such as vibrating systems, molds with special shapes (e.g., L-shaped), inclined chutes, or extrusion devices, allow precise control of the flow direction of the mixture and the orientation of the fiber [174,178,181,182,183,184,185]. The geometry of the mold and the position of the fibers relative to the wall of the formwork are also important. The wall effect, i.e., the limited possibility of fiber rotation at the edges of the mold, causes better orientation in the wall zone. Thinner samples are generally characterized by better fiber alignment due to a higher volume fraction of the wall effect zone [186]. Increasing the length of the casting mold leads to an extension of the zone in which the fibers are optimally arranged along the flow direction (so-called ordered flow zone) until the point of re-disruption is reached [187].

### 3.4. Fiber Hybridization

**Figure 2:**
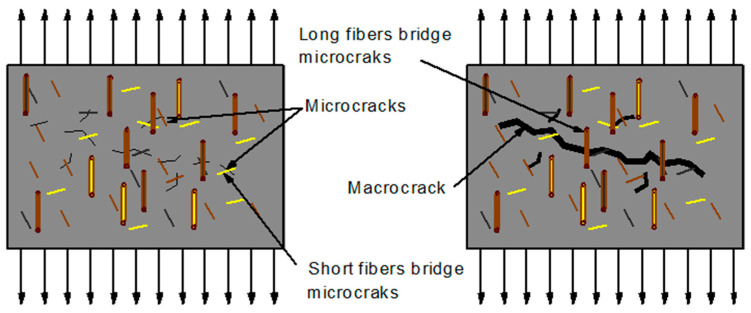
Schematic illustration of micro- and macrocrack bridging in UHPC by hybrid fibers.

Hybrid fiber is a composite material created by combining two or more types of fiber, allowing better properties to be achieved that cannot be achieved using a single type of fiber [188,189,190,191]. These fibers may vary in length, type, and shape, which affects their effectiveness in reinforcing concrete [64,192]. Mixing different types of fibers in a concrete matrix is considered an effective method to maximize their properties [21,193]. The introduction of fibers of different lengths promotes the creation of a continuous reinforcement network in concrete. Microfibers limit the development of microcracks, whereas macrofibers are responsible for controlling the formation and development of larger cracks (shown in Figure 2) [12]. Short fibers, evenly distributed in the cement matrix, prevent the initiation and propagation of microcracks, while longer fibers, due to the bridging effect, increase resistance to macrocracks [194,195,196,197]. Effective control of micro- and macrocracks results in increased strength of the composite material and a significant improvement in its resistance to cracking [50,198]. Due to this synergistic mechanism, the ductility of the material is largely determined by the presence of long fibers [199]. Different types of fiber have unique properties that can complement each other, compensating for the strengths and weaknesses of each other [12]. A single type of fiber improves the mechanical properties and durability of the material but is not effective in preventing the formation of large cracks. The hybridization of fibers allows for multilevel fracture mechanisms to be obtained. For example, low-modulus fibers increase ductility, while high-modulus fibers improve strength and stiffness, which, in combination, give optimized material properties [188]. The use of both types of fibers in concrete provides numerous benefits—low-modulus fibers improve the workability of the mixture, prevent cracking in the initial phase of concrete maturation, and increase its ductility [188], while high-modulus fibers are responsible for greater strength and microcrack-bridging effect. Fibers of different elasticity are often used in cement composite reinforcement; those with greater stiffness provide high strength and first crack stress, while more flexible fibers improve the ability to deform after cracks occur.

Recent research indicates that the hybridization of fibers in a cement composite not only increases its ductility and improves its durability but also allows cost reduction compared to the use of a single type of fiber [198]. The introduction of fibers of different shapes further enhances the interaction between them, resulting in improved mechanical properties of fiber-reinforced concrete [12].

## 4. The Influence of Hybrid Fibers on the Mechanical Properties of UHPC

### 4.1. Steel Fiber Combinations

Table 3 summarizes the test results for the mechanical properties of UHPC with the addition of steel–steel fiber mixtures.

Akça and İpek [63] conducted research on the effect of hybrid steel fibers on the properties of ultra-high-performance concrete. In their experiments, they used cements of CEM 42.5 and CEM 52.5 and applied steel fibers of two different lengths: short and straight (SSF) 6 mm with a diameter of 0.20 mm and long and hooked (LHF) 35 mm with a diameter of 0.75 mm. The research was carried out on mixtures containing a total of 3% of the fiber volume, with a proportion of 2% of short fibers and 1% of long fibers. The research showed that the addition of hybrid steel fibers contributed to an increase in the compressive strength of concrete by 15 and 40% in cements of CEM 42.5 and CEM 52.5, respectively. Furthermore, the combination of fibers of different lengths improved the ductility of the concrete after reaching the maximum load and increased the ability of the material to bridge microcracks, positively affecting its mechanical properties. Tensile splitting strength increased by 12% and 26%, respectively, compared to the base concrete. The strong bond between the fibers and the UHPC matrix was observed to limit slippage of the fibers, which improved the mechanical performance of the concrete. When the effect of fibers on the modulus of elasticity of UHPC was analyzed, it was found that it systematically increased with increasing fiber content and increasing compressive strength. In contrast to traditional concretes, the use of hybrid fibers in UHPC contributed to a significant increase in stiffness, reaching a maximum modulus of elasticity of 62.5 GPa. Achieving such a high value was possible thanks to optimization of the mix composition, including reducing the water/cement ratio, the use of high-strength cement, and the appropriate selection of the fraction and type of aggregate.

Yu et al. [65] conducted experiments on ultra-high-performance concrete reinforced with steel fibers of different lengths and shapes. In the study, CEM 52.5R cement and three types of fibers were used: long hooked fibers (LHFs) of 35 mm length and 0.55 mm diameter, long straight fibers (LSFs) of 13 mm length and 0.20 mm diameter, and short straight fibers (SSFs) of 6 mm length and 0.16 mm diameter. The UHPC mixtures contained a total of 2% of the fiber volume in different configurations. The results showed that the use of hybrid reinforcement, which combines fibers of different lengths and shapes, significantly improved the mechanical properties of concrete. The best results were achieved with a combination of 1.5% hooked fibers (LHFs) and 0.5% short straight fibers (SSFs), which increased compressive strength after 28 days by 42% compared to control concrete. The highest flexural strength of 30.9 MPa was achieved for concrete with the same combination of fibers. In contrast, concrete containing only 2% of SSFs showed a lower flexural strength by approximately 30%. This was probably due to two factors. First, SSFs effectively bridged microcracks, whereas LSFs prevented the development of macrocracks. Second, the presence of LSFs improved the orientation of SSFs, limiting their rotation space, which resulted in a better distribution of fibers in the direction perpendicular to the load, thus improving the mechanical properties of concrete.

Türker et al. [60] focused on the analysis of the combination of short and long steel fibers and their effect on the compressive, flexural, and tensile strength of UHPC. In their study, they used two types of fiber: short straight steel fibers (SSFs) with a length of 13 mm and a diameter of 0.16 mm and long hooked steel fibers (LHFs) with a length of 60 mm and a diameter of 0.75 mm. The studies were carried out on a mixture containing 1% of short fibers and 0.5% of long fibers. The use of steel fibers contributed to the improvement in the mechanical properties of concrete. The compressive strength increased by 9% using a single type of fiber and by 13% in the case of the hybrid mixture compared to samples without fibers. The use of hybrid fibers did not have a significant effect on the modulus of elasticity. A 2% increase in modulus was observed for the combination of steel fibers, while the single type of fiber increased the modulus by 7%. The tensile strength of the beams without fibers was 4.8 MPa, while the use of single and hybrid fibers increased it to 9.32 MPa and 11.18 MPa, respectively. This means that the concrete with hybrid fibers had a 20% higher tensile strength compared to the mixture with a single type of fiber. Four-point bending tests showed that the effect of fibers on the cracking process varied depending on the serviceability limit state (SLS) and the ultimate limit state (ULS). In the SLS state, beams containing a single type of fiber showed a significant reduction in the number of cracks, and the use of fibers effectively limited the crack width compared to beams without fibers. On the other hand, hybrid beams did not achieve as good results because the long fibers present in the concrete matrix were not sufficiently activated to limit cracks. According to the authors, better results could be obtained by increasing the proportion of short fibers in the hybrid mixture. In the ULS condition, after exceeding the maximum load, the use of hybrid fibers effectively limited the crack width, especially at higher reinforcement ratios. This was due to the fact that most of the short fibers had already been removed, and the long fibers in the concrete matrix started to actively counteract the development of cracks.

Yoo et al. [51] reported the influence of straight, hook, and twisted fibers and their hybrid combinations on the properties of ultra-high-performance concrete. The tests included samples containing short straight steel fibers (SSFs) with a length of 19.5 mm and a diameter of 0.20 mm and long hooked steel fibers (LHFs) with a length of 30 mm and a diameter of 0.38 mm. The total volume of fibers in the mixtures was 2%. The results showed that the highest compressive strength, among the hybrid fiber samples, was achieved at 199 MPa for the samples containing 1.5% straight fibers and 0.5% hooked fibers. Compared to the sample with monofibers, there was a 10% decrease in strength. Straight fibers were found to be more effective in improving compressive strength than hook and twisted fibers, which can negatively affect the homogeneity of the material.

Park et al. [56] conducted studies on ultra-high-performance concrete reinforced with steel microfibers and steel macrofibers. The aim of their experiments was to determine the effect of fiber hybridization on the tensile strength and multiple fracture capacity of concrete. In the studies different types of steel fiber were used: long straight (LSF) of 30 mm length and 0.30 mm diameter, short straight (SSF) of 13 mm length and 0.2 mm diameter, long hooked (LHF) of 62 mm length and 0.775 mm diameter, short hooked (SHF) of 30 mm length and 0.375 mm diameter, and long twisted (LTF) of 30 mm length and 0.30 mm diameter. Concrete mixtures contained different combinations of fiber volumes, with macrofibers added at 1% and microfibers at 0.5%, 1.0%, and 1.5%. The addition of microfibers was found to have a beneficial effect on the hardening of deformation and the multiple cracking capacity of concrete, while increasing their content improved their tensile properties. The best results in terms of deformation hardening were obtained by the LTF and SSF mixture, reaching a tensile strength of 18.6 MPa, which is almost 52% higher than the sample without the addition of microfibers.

*Discussion*

Analysis of the research results showed that hybridization of steel fibers has a beneficial effect on the mechanical properties of ultra-high-performance concrete. The use of fibers of different lengths and shapes leads to an improved compressive, bending, and tensile splitting strength and also increases ductility and the ability to bridge microcracks.

The mechanical benefits of hybridizing steel fibers strongly depend on the proportion and geometry of each fiber type. For example, Yu et al. [65] showed that a mix of 1.5% long hook-end fibers and 0.5% short straight fibers yielded the best flexural performance, demonstrating that a dominant volume of long fibers complemented by a smaller amount of shorter ones may offer optimal synergy. However, as noted by Yoo et al. [51], an excessive fiber content can lead to poor fiber dispersion and increased porosity, which may reduce compressive strength. Therefore, maintaining a balanced volume fraction, generally not exceeding 2% of the total fiber content, with a long-to-short fiber ratio of approximately 3:1 appears to be beneficial to mechanical performance without significantly compromising workability.

The compressive strength of UHPC is increased by using hybrid fibers. In Akça and İpek [63], an increase of 15% and 40% was observed, depending on the cement type, while Yu et al. [65] obtained a maximum increase of 42% for a blend containing 1.5% long hook fibers and 0.5% short straight fibers. The high strength is due to the optimal arrangement of the fibers, which effectively bridges cracks. However, Yoo et al. [51] observed that in some cases, hybridization can slightly reduce compressive strength, which may be the result of uneven fiber distribution in the concrete matrix.

Flexural strength is also significantly improved by using hybrid fibers. Yu et al. [65] found that the best results were achieved with a mixture containing 1.5% long fibers and 0.5% short fibers, which allowed one to obtain a flexural strength of 30.9 MPa. This effect is due to the synergy between short fibers, which strengthen the concrete in the incipient cracking stage, and long fibers, which prevent further crack development. Similar results were obtained by Türker et al. [60]. However, in their studies, higher bending strength values were noted for concrete containing one type of fiber, which may suggest the need for better selection of proportions in the case of hybrid mixtures.

The combination of hybrid fiber also improves tensile strength. Park et al. [56] found that the addition of microfibers increased the ability of concrete to crack multiple times and harden. The best results were achieved for a mixture containing long wavy fibers and short straight fibers, for which the tensile strength was 52% higher than that of the concrete without fibers. Türker et al. [60] also observed an increase in this value to 11.18 MPa, which means a 20% improvement compared to concrete with single-type fibers.

The hybridization of fibers can also affect the elastic modulus of UHPC. Akça and İpek [63] showed a significant increase in modulus up to 62.5 GPa, while Türker et al. [60] observed only a small increase in the modulus of elasticity compared to concrete that contains only one type of fiber. In some cases, the modulus of elasticity of UHPC with hybrid fibers was comparable to the values obtained for single-fiber concrete, suggesting that its increase depends on the appropriate selection of the fiber combination.

Further research should focus on optimizing the proportion of fibers, analyzing the effect of their shape on the mechanical properties of UHPCs, and assessing the durability of this material under various environmental conditions. It is also worth developing numerical simulations and mathematical models that will allow for a more accurate prediction of the effect of different combinations of fibers on concrete properties. Another important research direction may be the use of composite fibers in hybrid reinforcement systems, which could further improve the UHPC strength parameters.

### 4.2. Combinations of Steel and Synthetic Fibers

Table 4 shows the test results of the mechanical properties of UHPC reinforced with mixtures of steel and synthetic fibers.

Bian et al. [66] conducted research on the effect of different types of fibers on the mechanical properties of concrete. Steel, basalt, and polyethylene fibers were used in the experiment. In ultra-high-performance concrete, CEM 42.5R cement mortar was used, to which 1.5% of the volume of fibers in different combinations was added. Steel fibers (SSFs) with a length of 13 mm and a diameter of 0.20 mm, basalt fibers (BSFs) with a length of 18 mm and a diameter of 0.024 mm, and polyethylene fibers (PeSFs) with a length of 18 mm and a diameter of 0.025 mm were used. The results showed that hybrid combinations of fibers are more effective in improving the mechanical properties of concrete than the use of single types of fiber. Mixtures with 1% steel fibers increased compressive strength by 22% compared to the control sample. The modulus of elasticity also increased by 9%. The best results were obtained thanks to the synergistic effect of the combination of steel and polyethylene fibers, which improved the stability of the fracture process and increased the overall ductility of concrete. In the sample containing all three types of fiber, the tensile strength increased by 38% compared to the control sample. Steel fibers were primarily responsible for the increase in strength, while PE fibers increased ductility and controlled crack development. A mixture of 0.5% steel fibers and 1% polyethylene fibers increased concrete fracture energy by 76% compared to samples containing only polyethylene fibers. The tests conducted confirmed that hybrid fiber combinations are more effective than the use of single types of fiber, especially compared to basalt fibers, which had a minimal effect on improving mechanical properties.

Researchers Smarzewski and Barnat-Hunek [58] conducted research on UHPC based on cement CEM I 52.5. In their experiments, they analyzed the effect of steel hook fibers (StSFs) and polypropylene straight fibers (PSFs) on the mechanical properties of concrete. The studies used mixtures containing 1% of the fiber volume in various combinations. Steel fibers were 50 mm long and 1 mm in diameter, while polypropylene fibers were 12 mm long and 0.025 mm in diameter. Concrete samples were prepared with coarse granite or granodiorite aggregate. The addition of fibers was shown not to have a significant effect on the compressive strength. However, a larger amount of polypropylene fibers was observed to cause a decrease in this strength. The reason was the more difficult dispersion of fibers in the mixture and the low modulus of elasticity of polypropylene. The highest compressive strength was achieved by concrete with granodiorite aggregate and 1% steel fibers. The addition of 0.25% polypropylene fibers caused a 7% decrease in compressive strength. In terms of tensile strength at splitting, the best results were achieved by concrete containing 1% steel fibers, which showed an increase in strength of 37% compared to concrete without fibers. In turn, the addition of 1% polypropylene fibers increased this strength by 14%. In the remaining cases, the use of polypropylene fibers reduced the tensile strength. In general, the results confirmed that the addition of fibers, regardless of their type and quantity, increased the splitting tensile strength compared to the concrete without reinforcement. Analysis of the modulus of elasticity showed that its value increased with a higher share of steel fibers. In the sample containing 1% of these fibers, the modulus of elasticity was 4% higher than in the control sample. In turn, the addition of 1% polypropylene fibers caused a 10% decrease in the static modulus of elasticity compared to the concrete without fibers. Tensile strength tests in bending showed that it depended on the amount and type of fibers added. The lowest values of this strength were recorded in mixtures containing only polypropylene fibers and in concrete without fibers. On the other hand, samples with hybrid fibers with 0.75% and 0.25% steel fibers achieved an increase of 17% and 10%, respectively, compared to the control sample. Regarding fracture energy, it was observed that as microcracks developed, steel fibers effectively limited the propagation of larger macrocracks. The highest maximum load value was obtained in the sample containing 0.5% steel and polypropylene fibers. This value was 38% higher than in the control sample. The studies showed that the addition of steel fibers significantly improved concrete tensile strength, fracture energy, and modulus of elasticity. Although polypropylene fibers did not significantly affect compressive strength, they improved tensile splitting strength and increased fracture energy. The combination of both types of fibers in the hybrid mixture improved the mechanical properties of the concrete, but the excessive share of polypropylene fibers weakened its strength. The results confirm that the addition of fibers significantly increases the ductility of the UHPC and reduces its brittle fracture.

Aisheh et al. [41] conducted research on ultra-high-performance geopolymer concrete. Their aim was to determine the effect of different combinations of fibers on the mechanical properties of concrete, such as compressive strength, tensile strength, bending strength, and modulus of elasticity. Research was carried out on samples containing straight steel fibers (StSFs) with a length of 15 mm and a diameter of 0.12 mm and straight polypropylene fibers (PSFs) with a length of 8 mm and a diameter of 0.033 mm. The mixtures contained a constant amount of PSF (0.25%) and different combinations of SSF, allowing for a review of the effect of these fibers on the mechanical properties of concrete. The research results show that with the increase in the content of steel fibers in the mixture, the compressive strength increased by 33% and 46%. The addition of polypropylene fibers improved the ductility of the concrete, but their excess led to a decrease in strength. The use of a hybrid mixture of SF (2%) and PPF (0.25%) improved the tensile strength by 1.9% compared to the concrete that contained only SF. However, the content of polypropylene fibers, when too high, weakened the mechanical properties of concrete, resulting from its lower strength compared to that of SF. The researchers also conducted tests on the effect of hybrid fiber reinforcement on flexural strength. They showed that the combination of steel and polypropylene fibers allowed an increase in this value by as much as 91% compared to the control mixture. Steel fibers were crucial to improving strength, while polypropylene fibers increased the ductility of concrete. The effect of fibers on the modulus of elasticity of concrete was also analyzed. The results of the tests showed that adding 0.25% polypropylene fibers to the mixture containing 2% SF resulted in a slight decrease in the modulus of elasticity by 1.24%. In general, studies showed that the appropriate proportions of steel and polypropylene fibers can lead to synergistic effects, improving both the strength and ductility of concrete. Excess amounts of polypropylene fibers negatively affected the mechanical properties, resulting from their lower strength compared to that of steel fibers.

Elsayed et al. [64] conducted research on the influence of different types of fibers on the properties of high-performance concrete and its behavior at high temperatures. The experiments used high-performance concrete with CEM I 42.5 N mortar and three types of fibers: steel hook fibers (StHFs) 50 mm long and 0.1 mm in diameter, polypropylene straight fibers (PSFs) 12 mm long and 0.018 mm in diameter, and glass straight fibers (GSFs) 12 mm long and 0.013 mm in diameter. The results of the research showed that steel fibers were the most effective in improving compressive strength, increasing it by 26% compared to control concrete. In addition, they effectively limited cracks and improved the residual strength of the concrete after exposure to high temperatures. The use of hybrid fiber combinations, especially the combination of steel with polypropylene or glass fibers, contributed to the improvement of tensile strength, especially at elevated temperatures. The presence of polypropylene and glass fibers had a significant effect on reducing the spalling of the concrete, due to their ability to control the pressure of water vapor within the cement matrix. However, their effect on mechanical properties after exposure to high temperature was less significant than in the case of steel fibers. HPC column tests showed that the use of hybrid fibers increased the resistance to bending, while concrete with only steel fibers was characterized by the highest residual strength after thermal exposure. The results obtained suggest that steel fibers play a key role in improving the strength and resistance of concrete after exposure to high temperatures. In turn, polypropylene and glass fibers are particularly effective in reducing damage during heating, which may be of significant importance in applications requiring resistance to exceptional loads.

Kang et al. [57] conducted research on the effect of different types of fiber on UHPC. In their experiments, they analyzed steel, basalt, and synthetic fibers, such as polyvinyl and polyethylene. The fibers tested had different lengths and diameters. The steel fibers were in two variants: short with a length of 16.3 mm and a diameter of 0.2 mm and long with a length of 19.5 mm and the same diameter. The basalt fibers were 12 mm long and had a diameter of 0.012 mm, while the polyvinyl fibers were the same length but with a larger diameter of 0.04 mm. Polyethylene fibers were 18 mm long and had a diameter of 0.012 mm. The aim of the investigation was to determine the effect of different combinations of fibers on the strength of UHPC, both in compression and in tension. The results showed that the hybridization of steel fibers with synthetic fibers caused a small 4% decrease in compressive strength compared to concrete containing only steel fibers. At the same time, the combination of steel fibers with synthetic fibers had a beneficial effect on the tensile properties of concrete, increasing its resistance to cracking and improving the ductility of the material. The best tensile properties were obtained in the mixture in which 33% of the steel fibers were replaced by PE fibers. These fibers were more effective in bridging microcracks than basalt and PVA fibers, indicating their important role in improving the ability of UHPC to deform under load. On the basis of the results of the study, the authors concluded that steel fibers remain a key element in increasing compressive strength, while synthetic fibers play an important role in crack-bridging mechanisms. They also suggested the need for further research on the fiber pullout and bridging mechanisms in hybrid concrete, which would allow an even better understanding of its behavior under load.

*Discussion*

Analysis of research results conducted by various authors indicates that the use of hybrid fiber combinations in high- and ultra-high-performance concretes significantly affects their mechanical properties. Studies by Bian et al. [66] showed that the combination of steel and polyethylene fibers improved the ductility of concrete and the stability of the cracking process. The highest tensile strength was obtained in the sample containing all three types of fiber, SSF, BSF, and PeSF, which confirms the synergistic effect of different fibers in the mixture. In turn, Smarzewski and Barnat-Hunek [58] noticed that the addition of steel fibers significantly improved the tensile strength and modulus of elasticity, while polypropylene fibers increased the energy of the fracture. Their research results confirmed that the hybrid combination of SSF and PSF fibers improved the mechanical properties of concrete, but the excess of polypropylene fibers could lead to a decrease in compressive strength. Research by Aisheh et al. [41] on ultra-high-performance geopolymer concrete showed that appropriate proportions of steel and polypropylene fibers can lead to synergistic effects, improving both the strength and ductility of concrete. The combination of 2% steel fibers with 0.25% polypropylene fibers allowed a significant increase in flexural strength. Elsayed et al. [64], who analyzed the effect of different fibers on the high-temperature properties of concrete, drew similar conclusions. Their results indicated that steel fibers significantly improved compressive strength, while polypropylene and glass fibers effectively reduced concrete spalling. Kang et al. [57] noted that hybridization of steel fibers with synthetic fibers can increase ductility and fracture toughness. In their study, the best tensile properties were achieved in a mixture in which 33% of the steel fibers were replaced by PE fibers. These fibers proved to be more effective in bridging microcracks than basalt and PVA fibers.

Studies conducted show that the use of a combination of fibers in HPC and UHPC allows one to obtain better mechanical properties than in the case of using a single type of fiber. Steel fibers remain the key component influencing compressive and tensile strength, while synthetic fibers (polyethylene, polypropylene, and polyvinyl) improve ductility and limit crack propagation. Basalt fibers had the least impact on improving the mechanical properties of concrete. The relationship between steel and synthetic fibers significantly influences the overall balance between strength enhancement and mix workability. Aisheh et al. [41] found that a combination of 2% steel fibers and 0.25% polypropylene fibers was effective in improving both strength and ductility. Similarly, Kang et al. [57] observed that replacing 33% of steel fibers with polyethylene (PE) fibers improved tensile behavior while reducing fiber agglomeration. These findings suggest that small additions of synthetic fibers, typically 10 to 20% of the total fiber volume, can enhance crack control and energy absorption while simultaneously mitigating workability issues associated with high steel fiber content. Further experiments should focus on optimizing the proportions of different types of fibers in the concrete mix. An important research direction may also be the analysis of the influence of fiber structure and geometry on crack-bridging mechanisms and the study of the behavior of hybrid reinforcement under extreme conditions, such as high temperatures or dynamic loads. Furthermore, it is worth conducting research on new types of synthetic fibers and their potential application in UHPC.

### 4.3. Combinations of Basalt and Synthetic Fibers

Table 5 summarizes the mechanical properties of concrete with different combinations of basalt and synthetic fibers.

Smarzewski [59] conducted research on the influence of basalt and polypropylene fibers on the properties of high-performance concrete. He analyzed the influence of these fibers both separately and in hybrid systems, examining their effect on compressive strength, tensile strength, and fracture energy. In the experiments, he used high-performance concrete based on CEM 52.5R cement mortar, enriched with basalt straight fibers (BSF) with a length of 12 mm and a diameter of 0.013 mm and polypropylene straight fibers (PSF) with a length of 12 mm and a diameter of 0.025 mm. The total fiber content in the mixtures was 1% by volume, and different combinations of these fibers were analyzed. Studies showed that the presence of hybrid fibers negatively affected the compressive strength of HPC. The largest decrease, of the order of 15–20%, was observed when basalt fibers were used alone, while the combination of polypropylene and basalt fibers resulted in a reduction in compressive strength of 11 to 18%. This phenomenon was explained by the reduced workability of the mixture and the formation of voids, which had a negative impact on the concrete structure. The author emphasized the need to improve the compaction process of the mixture to reduce these effects. In turn, analysis of tensile strength showed that the addition of hybrid fibers had a positive effect on this property. Tensile strength, using hybrid fibers, increased 40–45% compared to the control sample. Polypropylene fibers were found to be more effective than basalt fibers in improving this characteristic because they are better connected to microcracks due to their larger diameter, number, and smaller distances between fibers. However, increasing its content above 1% did not bring any significant benefits. Furthermore, studies on fracture energy showed that optimal results were achieved with lower fiber concentrations for hybrid mixtures, indicating the synergistic effect of different types of fiber in increasing resistance to concrete fractures. Studies have shown that although hybrid fibers can reduce compressive strength, they also improve tensile strength and fracture energy. Polypropylene fibers were found to be more effective than basalt fibers in increasing tensile strength and fracture toughness, while their combination with basalt fibers gave the best results at lower concentrations.

Deng et al. [62] conducted research on the effect of different types of fibers on the flexural strength of concrete. The tests were carried out on samples of ultra-high-performance concrete with two types of fiber reinforcement: 19 mm long straight basalt fibers with a diameter of 0.013 mm and 50 mm long twisted polypropylene fibers (PTFs) with a diameter of 0.8 mm. The test results showed that the control sample without fiber addition achieved a flexural strength of 4 MPa. The introduction of basalt fibers increased strength by 10%. Even better results were obtained in the case of concrete with the addition of polypropylene macrofibers, where the strength increased by 30%. The highest flexural strength was observed in the sample containing a hybrid combination of basalt and polypropylene fibers in a mass ratio of 1:2. In this case, the strength increased by 42.5% compared to the control sample. The tests showed that polypropylene macrofibers have a greater effect on improving flexural strength than basalt fibers. The mechanism of action of hybrid fibers in this combination is that basalt fibers limit the development of microcracks in concrete, but their low deformation capacity means that they do not significantly improve its brittleness. On the other hand, polypropylene macrofibers play a bridging function, distributing stresses and increasing the concrete’s resistance to the formation of macrocracks.

Wang et al. [61] conducted research on the effect of basalt and polypropylene fibers on the mechanical properties of HPC. In the production of concrete, the CEM 42.5 cement class was used, to which two types of fiber were added: basalt straight fibers (BSFs) with a length of 12 mm and a diameter of 0.012 mm and polypropylene straight fibers (PSFs) with a length of 12 mm and a diameter of 0.02 mm. Their effect on compressive strength, bending strength, and tensile strength at splitting was analyzed. Research results showed that the addition of fibers affected the compressive strength of HPC depending on their volume fraction. With a basalt fiber fraction of 0.1% and 0.2%, the addition of polypropylene fibers led to a decrease in compressive strength, which was caused by the agglomeration of fibers and the formation of weak interfaces in the concrete structure. The optimal compressive strength was obtained with a basalt fiber fraction of 0.15% and a polypropylene fiber fraction of 0.033%. In this configuration, the strength increased by 14% compared to HPC without fibers. The best results in flexural strength were obtained for concrete containing 0.15% basalt fibers and 0.025% or 0.033% polypropylene fibers. In these cases, the flexural strength increased by 26% and 23%, respectively, compared to HPC without fibers. When the basalt fiber fraction was increased to 0.2%, the flexural strength fell below the values obtained for HPC with a single type of fiber, but it was still higher than for concrete without fibers. The analysis showed that polypropylene fibers have a greater effect on improving flexural strength than basalt fibers. The results of the splitting tensile strength tests showed that the use of both types of fibers led to an increase compared to that of the concrete without fibers. The best value was obtained for concrete containing 0.15% basalt fibers and 0.025% polypropylene fibers. In this combination, the tensile strength increased by 52% compared to the base sample. When the basalt fiber content was increased to 0.2%, the strength decreased, but was still higher than the HPC without fibers. Studies showed that appropriate proportions of basalt and polypropylene fibers can significantly improve the mechanical properties of HPC. The best results were obtained with a basalt fiber fraction of 0.15% and a polypropylene fiber fraction in the range of 0.025% to 0.033%. Such combinations had a positive effect on the strength properties. Excess amounts of basalt fibers led to agglomeration and weakening of the concrete structure, resulting in a deterioration of its mechanical properties.

*Discussion*

Tests Smarzewski [59], Deng et al. [62] and Wang et al. [61] showed that the use of a combination of basalt and polypropylene fibers in high-performance and ultra-high-performance concrete can have both positive and negative effects on mechanical properties. In relation to compressive strength, it was observed that the addition of hybrid fibers leads to its reduction. Smarzewski [59] found that the greatest reduction of 15 to 20% occurred when basalt fibers were used alone, while their combination with polypropylene fibers resulted in a smaller reduction of 11 to 18%. Wang et al. [61], who observed that inappropriate fiber proportions can lead to agglomeration and the formation of weak interfaces in the concrete structure, obtained similar results. In the case of the combination of basalt and synthetic fibers, studies have shown that the right selection of their proportions is crucial. Wang et al. [61] showed that the optimal increase in tensile and flexural strength was obtained using 0.15% basalt fibers and 0.025 to 0.033% polypropylene fibers, indicating that even small amounts of fibers can generate strong synergistic effects. However, exceeding these proportions led to a decrease in compressive strength and increased the risk of fiber agglomeration. Therefore, a balanced mass ratio of basalt to polypropylene fibers of around 1:2 is considered particularly effective, especially in the context of minimizing the negative impact on the compaction and porosity of the mixture.

However, when the optimal fiber ratio (0.15% basalt and 0.033% polypropylene) was used, the compressive strength increased by 14%. In turn, tensile strength improved significantly as a result of the use of hybrid fibers. Smarzewski [59] showed that their addition increased this value by 40–45% compared to the control sample. Polypropylene fibers were more effective in improving this property than basalt fibers, which was due to their better distribution and their ability to limit microcracks. Wang et al. [61], who reported similar results, observed that the highest tensile strength was obtained with 0.15% basalt fibers and 0.025% polypropylene fibers. A positive effect of hybrid fibers was also observed in the case of bending strength. Deng et al. [62] showed that the highest strength was obtained for a mixture containing basalt and polypropylene fibers in a mass ratio of 1:2. In this case, the strength increased by 42.5% compared to the control sample. Wang et al. [61] confirmed these results, indicating that optimal proportions of fibers (0.15% basalt and 0.025–0.033% polypropylene) increased flexural strength by 23–26%. Another important issue is the effect of hybrid fibers on fracture resistance. Studies have shown that their use significantly improves this property. Basalt fibers limit the development of microcracks, while polypropylene fibers, especially macrofibers, play a bridging function, transferring stresses, and increasing the ability of concrete to absorb energy. Due to this, the hybrid combination of fibers contributes to limiting the propagation of cracks and increasing the durability of concrete.

In summary, the use of hybrid fibers in high-performance concrete brings both benefits and certain limitations. The biggest problem is the reduction in compressive strength, resulting from difficulties in compacting the mixture and the formation of voids. In terms of tensile strength, bending strength, and fracture toughness, the combination of basalt and polypropylene fibers shows synergistic effects, leading to significant improvement in these parameters. Therefore, further research should focus on optimizing the proportion of fibers in concrete mixtures to minimize the negative impact on compressive strength while maximizing benefits in terms of other mechanical properties. It is also important to develop methods to improve the workability of the concrete and compact the mixture, which would reduce porosity. It is also worth conducting analyses of the effect of fiber geometry, comparing the effectiveness of straight, twisted, and wavy fibers. Another direction of research should be the evaluation of the long-term durability of concrete with hybrid fibers, particularly in the context of resistance to weather conditions, material fatigue, and degradation processes. In addition, it is worth using numerical modeling to predict the effect of different combinations of fibers on concrete properties and design cement mixtures more effectively with improved parameters.

### 4.4. Summary of Mechanical Property Test Results

#### 4.4.1. Compressive Strength

**Figure 3:**
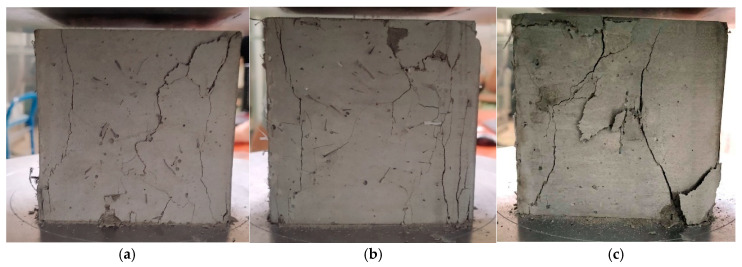
Compressive failure modes of UHPC with different hybrid fiber content: (**a**) 0.75% polyolefin 54 mm + 0.75% B 12 mm, (**b**) 0.75% polyolefin 54 mm + 0.75% PP 12 mm, (**c**) 0.75% PP 38 mm + 0.75% B 12 mm.

Figure 3 shows samples of ultra-high-performance concrete reinforced with different combinations of hybrid fibers destroyed by compression. In a concrete cube made of ultra-high-performance concrete without the addition of fibers, the destruction mechanism is typical of brittle materials. After the maximum compressive stress is exceeded, a sudden and abrupt destruction occurs, which leads to a rapid disintegration of the sample into many fragments. However, fibers act as bridges in microcracks, limiting their development and slowing the propagation of cracks. Instead of sudden disintegration of the sample, a gradual cracking is observed, and the material structure can transfer loads for a longer time. The destruction due to compressive stress in samples with non-metallic fibers with a total content of 1.5%, including longer fibers (54 or 38 mm) and short fibers (12 mm), is shown in Figure 3a–c. The crack arrangement was quite regular, and, in some places, a network of small cracks was formed (Figure 3a,b). A larger number of short PP fibers than B fibers in the sample volume resulted in an increase in the number of cracks with smaller widths. However, the combination of long PP fibers and short B fibers (Figure 3c) caused a further reduction in the total number of cracks and an increase in their width due to the wavy shape of the PP fibers. The debonding of a part of the concrete in the corner and on the surface indicates a very good anchoring of the wavy PP fibers, which is manifested by the chipping of fragments of the sample during the straightening of fibers located in the zones near the surface.

**Figure 4:**
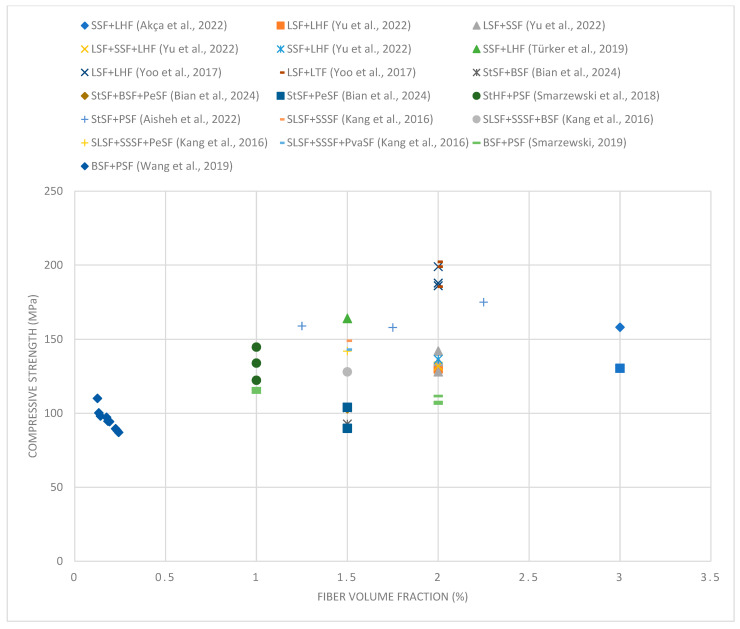
Effect of hybrid fiber combinations on compressive strength.

Figure 4 shows that the compressive strength increases with increasing volume fraction of fibers to a level of about 1.5–2%. Beyond this value, increasing the amount of fibers no longer brings significant benefits. Excess amounts of synthetic fibers, such as polypropylene fibers, can cause a decrease in compressive strength. This happens for several reasons. First, a large volume of synthetic fibers can disrupt the homogeneity of the concrete mix, making it difficult to compact it properly, which leads to the formation of voids and local weakening of the structure. Second, synthetic fibers, characterized by lower stiffness and strength compared to steel or basalt fibers, can replace the active participation of the concrete skeleton in transferring loads in excessive amounts. As a result, the structural continuity of the concrete is weakened, and its load-bearing capacity under compression is reduced. In addition, an excess of synthetic fibers can increase the tendency of the mix to segregate and hinder the process of cement–aggregate bonding, which also results in a decrease in mechanical parameters [12,140].

The best results were achieved for mixtures containing steel and basalt fibers, indicating their beneficial effect on the structure of concrete. However, combinations of long straight steel fibers and twisted fibers did not show such high strength, suggesting that the presence of long fibers alone is not sufficient to obtain maximum benefits. Combinations containing short straight steel fibers and long hook-end fibers show moderate strength, suggesting that the combination of fibers of different shapes can effectively improve mechanical properties. In the case of mixtures with polymer fibers, their addition has a slight effect on the increase in compressive strength but may improve other parameters of the concrete, such as fracture toughness.

Compared to traditional fiber-reinforced concretes, UHPC with hybrid fibers shows much higher compressive strength. While typical compressive strengths of FRC range between 30 and 60 MPa, the UHPC mixtures tested in this study reach values greater than 120 MPa. This significant increase results from the dense matrix and effective crack-bridging mechanisms provided by hybrid fibers, which improve post-peak behavior and delay catastrophic failure, making UHPC more suitable for high-load structural applications.

#### 4.4.2. Tensile Strength

**Figure 5:**
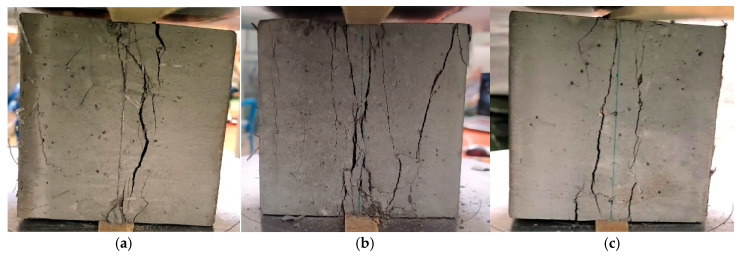
Tensile splitting failure modes of UHPC with different hybrid fiber content: (**a**) 0.75% polyolefin 54 mm + 0.75% PP 12 mm, (**b**) 0.75% polyolefin 54 mm + 0.75% B 12 mm, (**c**) 0.75% PP 38 mm + 0.75% B 12 mm.

Figure 5 shows different failure modes of UHPC samples after splitting tensile tests. The failure mechanism of the UHPC cube without added fibers is characterized by a sudden splitting of the sample. After reaching the limit stress, it cracks in the middle of the cross-section, with a clear separation into two main parts, completely losing its load-bearing capacity. The influence of the type of fiber is observed in Figure 5a,b in the comparison of failure modes of two concretes with the addition of 0.75% polyolefin fibers 54 mm and 0.75% PP fibers 12 mm or 0.75% B fibers 12 mm. In the case of the mixture with two types of polymer fibers (see Figure 5a), one main crack is observed running along an arc, indicating the loss of load-bearing capacity by the matrix and the taking over of some of the residual loads by the fibers. A network of vertical microcracks is also noticeable, indicating the absorption of some additional portion of energy during cracking. The same addition of B 12 mm fibers replacing PP 12 mm fibers resulted in the formation of a greater number of main vertical microcracks running through the entire cross-section of the sample, which indicates the involvement of an additional part of the matrix together with a portion of fibers in load transfer and a higher capacity for energy absorption. The replacement of the same content of long polyolefin fibers with wavy PP fibers (Figure 5c) while leaving 12 mm B fibers resulted in the formation of two main cracks of smaller width and few vertical microcracks. This indicates that PP fibers absorbed significantly higher fracture energy than longer polyolefin fibers. This can be explained by significantly better anchoring of wavy PP fibers in the UHPC matrix. In all of the cases presented, the fibers prevented complete separation of the sample and, through the action of holding forces, delayed the propagation of cracks and reduced the width of their opening but, at the same time, caused the appearance of UHPC crushing spots under and above the spacers.

**Figure 6:**
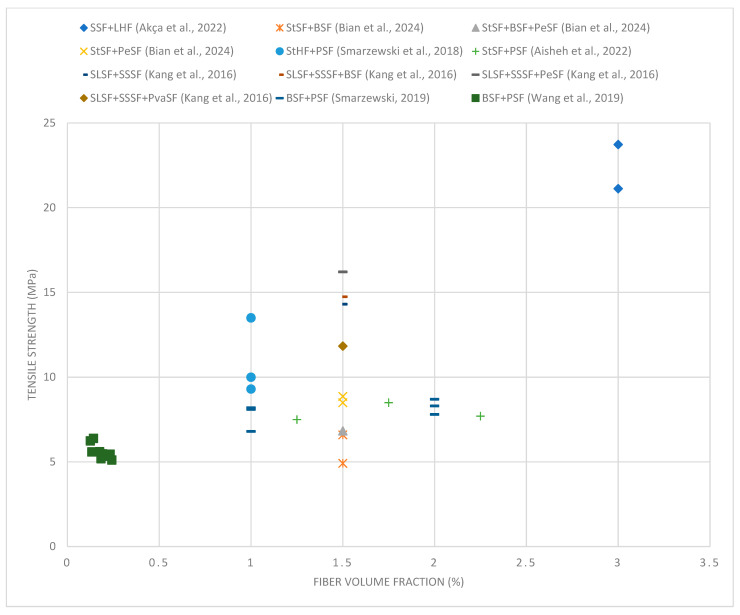
Effect of hybrid fibers on tensile strength of UHPC.

The analysis of the results presented in Figure 6 shows that different combinations of hybrid fibers have different effects on tensile strength. The highest strength, about 22 MPa, is achieved by the combination of short straight fibers with long hook fibers at a volume fraction of 3%. This means that the use of long hook fibers significantly improves the tensile strength of the material. Furthermore, the combination of steel hook-end fibers with polypropylene fibers at a volume fraction of 1% shows a relatively high strength of about 12 to 14 MPa. Combinations containing steel fibers in combination with basalt or polyethylene fibers, such as StSF + BSF, StSF + BSF + PeSF, and StSF + PeSF, show a smaller effect on improving tensile strength, remaining in the range of 5 to 10 MPa at a content of 1.5%. Increasing the fiber volume fraction does not always result in an increase in strength. For example, for the combination BSF + PSF, increasing the volume fraction above 1.5% does not bring any further improvement in strength, which remains at a level of 5–7 MPa. In contrast, SSF + LHF is the only combination that shows a significant increase in strength with increasing fiber fraction, which may be due to the synergistic effect of short straight and long hook fibers in effective stress redistribution.

Compared to traditional FRC, which usually shows tensile strength values in the range of 2 to 6 MPa, the UHPC samples reinforced with hybrid fibers exhibit substantially enhanced performance, reaching up to 22 MPa. This increase is primarily due to the synergistic interaction of fibers with varying lengths and geometries, which provides better stress redistribution and more effective crack control. This makes UHPC with hybrid fibers particularly advantageous in structural elements exposed to tension or requiring high ductility, such as beams, slabs, or seismic components.

#### 4.4.3. Flexural Strength

**Figure 7:**
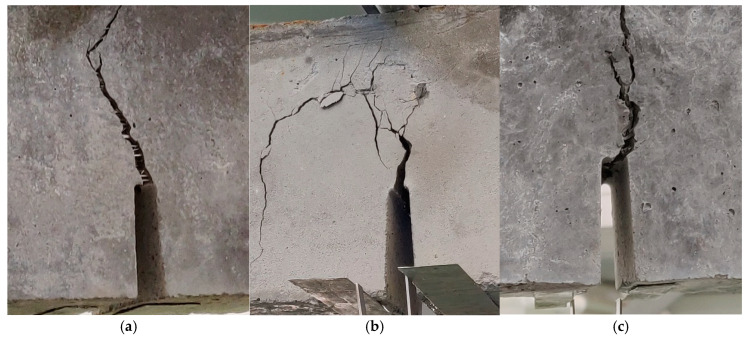
Flexural failure modes of UHPC with different hybrid fiber content: (**a**) 1.5% S 50 mm + 1.5% G 18 mm, (**b**) 1.5% S 50 mm + 1.5% B 12 mm, (**c**) 1.5% B 12 mm + 1.5% PP 40 mm.

Figure 7 shows notched UHPC samples with hybrid fiber addition, which failed under bending load. The UHPC sample without fiber addition, after reaching the limit stresses, suddenly breaks in the middle of the cross-section, completely losing its ability to transfer load. In the case of samples with fiber addition, the crack propagates from the tip of the notch to the upper surface of the sample, undergoing multiple refractions. In the case of the sample with an addition of 1.5% S and 1.5% G, steel and glass fibers are visible in the middle of the damaged cross-section (Figure 7a), which maintains the UHPC structure against complete cracking. The lack of microcracks is caused by the high content of short G fibers, which have a low density and small diameter and, therefore, occur in a much greater number than hooked steel fibers. They take on the stresses that arise until their load-bearing capacity is exceeded, after which the additional load is transferred by the hooked steel fibers. In Figure 7b, one can see completely different patterns of branched cracks formed in the beam with the addition of 1.5% S and 1.5% B. The crack formed at the tip of the notch does not propagate upward but changes direction to horizontal. The high tensile strength of the fibers and the high forces that hold them in the matrix do not allow the development of vertical cracks. Additional microcracks indicate a lower effectiveness of basalt fibers in inhibiting them compared to glass fibers. The sample with the addition of 1.5% B and 1.5% PP (see Figure 7c) has visible uneven edges and microcracks at the location of the main crack, which may indicate an effective influence of wavy PP fibers on the processes of inhibiting crack propagation due to very good anchoring in the matrix and a large portion of energy needed to straighten the fibers during their pulling.

**Figure 8:**
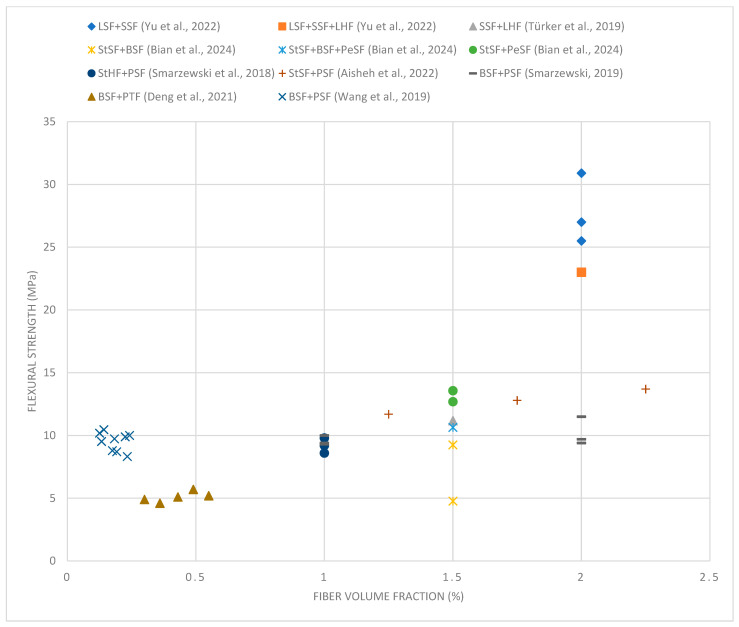
Influence of hybrid fibers on UHPC flexural strength.

Analyzing the results in Figure 8, it can be seen that hybrid fiber combinations have a different effect on the bending strength. The combinations of steel fibers with synthetic fibers [41,58,66] and the combinations of basalt fibers with synthetic fibers [59,61,62] show relatively low bending strength at low values of the fiber volume fraction (approximately 1%). On the other hand, combinations of steel fibers with steel fibers [60,65] achieve higher strength values, especially at higher fiber volume fractions (approximately 2%). This means that the presence of steel fibers in both fractions can have a positive effect on the bending strength, while basalt fibers with synthetic fibers do not increase it to such an extent.

In structural applications requiring flexural capacity, UHPC with hybrid fibers also outperforms conventional FRC. Traditional FRC often shows limited flexural strength and fails in a brittle manner after cracking. In contrast, hybrid fiber-reinforced UHPC shows deflection-hardening behavior, with the ability to carry load beyond the first crack and sustain multiple crack formations. This performance advantage enables the design of thinner and lighter elements while ensuring safety and serviceability under bending loads.

#### 4.4.4. Elastic Modulus

**Figure 9:**
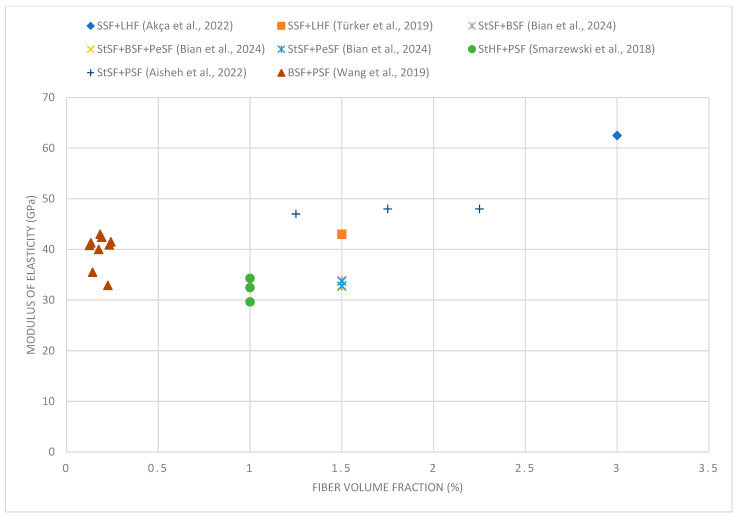
Influence of hybrid fibers on modulus of elasticity.

Analysis of the combination presented in Figure 9 shows that the modulus of elasticity depends on the type of hybrid fibers used and their content. The combinations of steel fibers with synthetic fibers [41,58,66] show different values of the modulus of elasticity, but most are in the range of 30 to 50 GPa. In turn, the combination of steel fibers with steel fibers [60,63] reaches an elastic modulus of about 40 GPa, which is similar to some results obtained for steel–synthetic combinations. In the case of the combination of basalt fibers with synthetic fibers [61], the modulus of elasticity values are lower, which suggests that the presence of basalt fibers may reduce the stiffness of the material in comparison to the variants with steel fibers. In general, the best results in terms of the modulus of elasticity were obtained for the combination of straight short steel fibers with long hooked fibers, especially at 3% fiber content. In addition, the combination of basalt and polypropylene fibers shows a high value of the modulus of elasticity. It is worth noting that the modulus of elasticity does not always increase with increasing fiber content; the appropriate selection of their type and proportions is crucial.

Although both UHPC and traditional FRC can achieve similar ranges of elastic modulus (typically 30–50 GPa), UHPC with appropriately selected hybrid fibers maintains high stiffness while offering enhanced toughness and crack resistance. This balance is particularly valuable in elements that require minimal deformation under service loads, such as precast bridge components or façade panels.

#### 4.4.5. Fracture Energy

**Figure 10:**
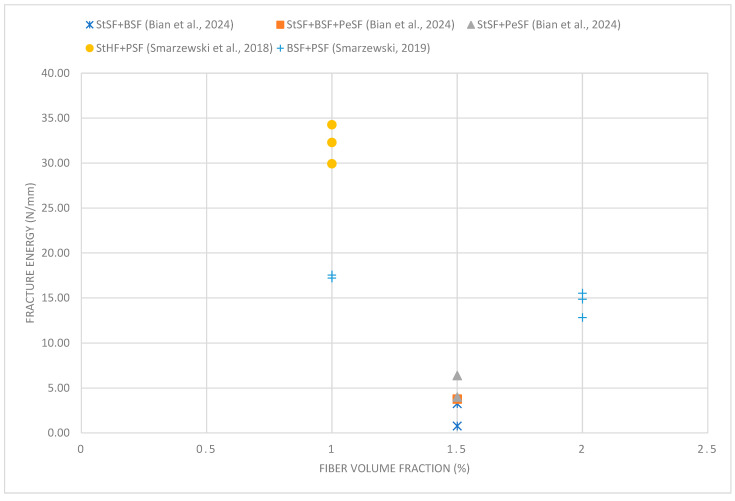
Influence of hybrid fibers on fracture energy.

When analyzing Figure 10, it can be seen that different combinations of hybrid fibers have a significant effect on the fracture energy. The highest energy value was achieved for the sample containing a combination of steel-hooked fibers and straight polypropylene fibers at 1% of the fiber volume. This result suggests that the combination of these two types of fibers shows a synergistic effect, significantly improving the mechanical properties of the material. The addition of basalt fibers in different combinations did not result in a significant increase in fracture energy. Examples such as StSF + BSF and BSF + PSF indicate that, particularly, short basalt fibers have a limited effect on improving fracture resistance. Similarly, the sample containing steel straight fibers and polyethylene straight fibers at 1.5% of the fiber volume content showed a lower fracture energy than StHF + PSF. This may suggest that both the geometry and the type of synthetic fibers play a key role in shaping the mechanical properties of the material.

A key advantage of UHPC with hybrid fibers over traditional FRC lies in its significantly higher fracture energy. While conventional concretes often exhibit brittle failure with limited energy dissipation, UHPC can absorb and redistribute much larger amounts of energy due to the interaction of fibers during crack propagation. This is especially relevant in applications that require high impact or blast resistance, where energy absorption is critical for structural integrity.

### 4.5. Summary of Key Findings from the Mechanical Parameter Review

Studies on the mechanical properties of UHPC concrete with the addition of hybrid fibers have shown a significant effect of the type, shape, and proportion of fibers on improving the strength of the material. Concrete without fibers is characterized by brittle behavior and rapid destruction after exceeding the load-bearing limit. The addition of hybrid fibers, especially the combination of short straight and long hook steel fibers, allows for effective dispersion and inhibition of crack propagation, which significantly increases compressive, tensile, and bending strength. Table 6 summarizes the highest values of the strength parameters of the studies discussed above, taking into account the type of hybrid fiber. The best results are achieved with a volume fraction of fibers at the level of 1.5–2%. In terms of tensile strength, the use of the SSF + LHF mixture in the amount of 3% allowed for achieving exceptionally high strength, up to 22 MPa, which is several times higher than in traditional concrete reinforced with fibers. In the case of bending, the combination of steel fibers with glass fibers is also effective, ensuring the development of numerous small cracks instead of one dominant crack. In terms of the modulus of elasticity, the highest stiffness is provided by mixtures with steel fibers, although some systems with basalt and synthetic fibers also showed high values. The key conclusion is that not the quantity itself but the proper selection and differentiation of fibers—in terms of length, shape, and material—determine the effectiveness of reinforcement and improvement of mechanical properties of UHPC concrete.

Despite significant progress in research on the effect of hybrid fibers on the mechanical properties of UHPC, there is still a noticeable lack of standardized test methods that would take into account the specificity of such blends. Existing standards usually refer to single types of fiber but do not sufficiently cover their combinations, the mechanical effects of which can be synergistic and difficult to capture within the framework of classical procedures.

Therefore, it is necessary to develop integrated research protocols, including both classical tests and advanced measurements of fracture toughness, fatigue, and degradation under long-term loads. This approach should also assume a comparison of materials with appropriately selected references.

Analogous standardization needs also appear in other areas of materials research. In the work by Njimou et al. [198], with respect to the AlOP bio-nanocomposite, a comprehensive physicochemical and electrochemical characterization of the newly developed material was carried out, comparing its properties with the reference material. Thanks to the use of a multi-step approach, it was possible to reliably confirm its functional properties, which is also a good methodological model for the evaluation of UHPC with hybrid fibers.

## 5. Durability of UHPC with Hybrid Fibers

Concrete durability tests assess its resistance to degradation factors, such as frost, de-icing salts, carbonation, chemicals, and high temperatures [1]. The aforementioned tests are important to determine their suitability under operating conditions. They allow the prediction of the behavior of concrete in the long term of use and enable the selection of appropriate mixture components and maintenance methods to ensure the longevity of the structure. One of the basic tests is the analysis of concrete water absorption and porosity, which allows for the assessment of the ability of the material to absorb water and susceptibility to chemical corrosion. Other important tests are the following.

- Frost resistance test, consisting of cyclic freezing and thawing of samples in a water or salt solution and then assessing their strength.
- Carbonation test, i.e., the process of carbon dioxide penetrating the material’s structure, which leads to a decrease in pH and may result in corrosion of the reinforcement.
- Chloride resistance test, consisting of exposing a concrete sample to a chloride solution and then analyzing the degree of penetration and diffusion coefficient.
- Fire resistance test, e.g., by subjecting the sample to controlled heating according to standard temperature curves and then analyzing its residual strength and degree of degradation.

### 5.1. Corrosion Resistance

Corrosion resistance tests allow the durability of concrete to be evaluated under difficult operating conditions and help optimize its composition. Permeability and salt resistance tests are particularly important.

#### 5.1.1. Permeability Test

The permeability of concrete refers to the ability of a material to pass through fluids, including water. As a result of the high permeability of water through concrete, various chemicals, such as chloride ions, can penetrate the material, causing corrosion of the reinforcement bars or fibers. Properties such as the water–cement ratio, the type of additional cementitious materials, the diameter of the pores, and their connectivity have a significant effect on the permeability of concrete [200,201,202,203,204,205,206,207]. In studies of the water permeability coefficient of different types of concrete, the UHPC showed a permeability coefficient of around 0.0005 after 98 days, which was due to the extremely low porosity of this material. In comparison, traditional concrete achieved a permeability coefficient of around 0.0015 after 98 days, which is an order of magnitude higher. This difference can be explained by the more homogeneous, compact, and dense structure of cement paste in UHPC, where small and discontinuous pores dominate [208,209,210,211].

Li et al. [67] indicated that hybrid fibers can prevent the formation of microcracks in concrete and also improve its waterproofing. Xu et al. [69] confirmed that hybrid fibers have a positive effect on the waterproofing of concrete, and their effectiveness is more evident with a lower content of steel fibers in the mixture. In turn, Yang et al. [69] noted that hybrid fibers can have a positive and negative effect on the waterproofing of concrete. Too many of them can reduce the compactness of the material, leading to enlargement of the internal pores and weakening of the structure, which results in poorer concrete waterproofing. Liu [70] showed that the best results in terms of waterproofing are achieved with a hybrid fiber content of 0.05% to 0.1%, and their excess (more than 0.2%) causes deterioration of the waterproof properties.

**Figure 11:**
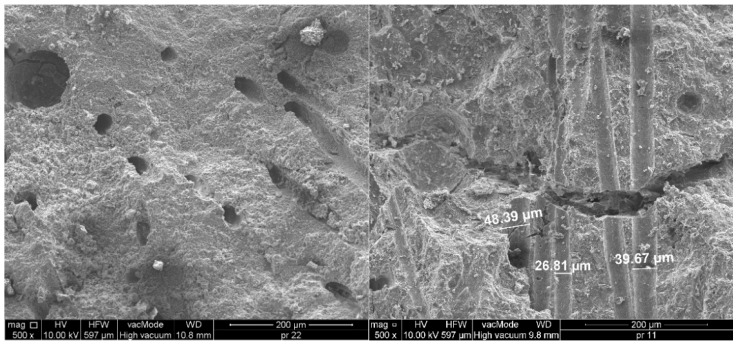
Microchannels created by melting PP fibers at temperatures above 600 °C and 800 °C.

The research by Hung [71] showed that the depth of permeability of UHPC samples at ambient temperature ranged from 5 to 11 mm. A higher PP fiber content was observed to slightly increase the depth of permeability. At 100 °C, the depth increased to 19–26 mm, suggesting an increase in micropores due to thermal effects and the decomposition of ettringite. With a further increase in temperature to 200 °C, the permeability increased significantly, and the sample with 1.5% steel fibers and 1% PP fibers showed a depth of permeability of up to 55 mm. X-ray studies indicated that the increase in permeability was due to portlandite decomposition at approximately 200 °C, which led to the formation of lime and water vapor and, consequently, to an increase in porosity and internal pressure. Additionally, the higher permeability of UHPC samples with hybrid fibers suggests that microcracks formed as a result of high temperatures forming connections with the microchannels of the fibers, which increases the permeability of these samples. The results of Hung [71] also indicate that although higher temperatures generally increased the water absorption rate in UHPC samples, all variants maintained it below 3% up to 300 °C. The inclusion of PP fibers had no significant effect on the water absorption rate or increased it only slightly. However, significant differences were observed above 300 °C. Steel fiber samples without PP additions suffered from severe explosive spalling and could not be tested above this temperature due to loss of integrity. In contrast, the samples containing 0.5% and 1% PP fibers and steel remained largely intact. This demonstrates the effectiveness of hybrid fibers in reducing UHPC spalling by facilitating the release of water vapor pressure trapped in the concrete pores through microchannels formed by the melting of PP fibers. Figure 11 shows photos of the microstructure of UHPC with the addition of hooked steel fibers and straight PP fibers after thermal exposure.

Studies have shown that both the type of concrete and the content of hybrid fibers have a significant impact on the permeability of water, which directly translates into the resistance to corrosion of concrete. The high porosity of traditional concrete promotes the migration of chemicals, while materials such as UHPC, characterized by lower porosity and better compactness, show greater resistance to water penetration. Additionally, the presence of hybrid fibers can improve the water tightness of concrete, but their excess can have an adverse effect, increasing porosity and weakening the structure. At elevated temperatures, hybrid fibers play a key role in reducing the spalling of UHPC, improving its durability and stability in extreme conditions.

#### 5.1.2. Resistance to Chloride and Salt Penetration

Corrosion of the reinforcement steel in concrete, caused by the penetration of chloride ions, is one of the key threats to the durability of concrete structures. Ultra-high-performance concrete is characterized by an extremely low permeability to chloride, which significantly increases its corrosion resistance [28]. Chloride ions can diffuse through concrete in two ways: as free ions dissolved in the pore solution or as chemically and physically bound to the hydration products [40,41,42,212,213]. Free chloride ions can cause the depassivation of reinforcement steel and steel fibers, initiating a corrosion process that causes the degradation of concrete structures [214,215]. In turn, the high alkalinity of the pore solution in UHPC supports the passivation of the steel, which delays the initiation of corrosion.

Numerous studies have been conducted to assess the degree of protection provided by UHPC to steel reinforcements. Ghafari et al. [73] performed an accelerated corrosion test on steel bars embedded in HPC and UHPC, analyzing the degradation rate. The results indicated that the time to the initiation of corrosion in UHPC was more than twice as long as in HPC. Furthermore, the measured corrosion rate was only 0.01 mm/year, which is well below the limit value of 1 mm/year [216]. The evaluation of chloride ion permeability in UHPC is a key aspect of the analysis of its corrosion resistance. For this purpose, accelerated test methods are often used, such as the rapid chloride permeability test and the Nordtest NT Build 443 method [28]. In particular, the latter method uses the chloride ion diffusion coefficient as an indicator to assess the resistance of UHPCs to the penetration of these ions. A comparison of the results of the chloride permeability test in UHPC presented in different publications shows a wide range of diffusion coefficient values from 1.3 × 10^−13^ m^2^/s to 4.1 × 10^−13^ m^2^/s [217,218,219]. These values depend on the water/binder ratio (W/B), the curing method, the chloride concentration in the exposure environment, the steel fiber content, and the age of the samples during testing. The difficulty in accurately comparing the resistances of different UHPCs to chloride penetration is due to differences in the parameters of the test procedures used and the proportions of the mixtures analyzed in the literature. However, it should be noted that the chloride ion diffusion coefficient in UHPC is at least an order of magnitude lower than in conventional and high-performance concretes [217,218,219]. This shows the high resistance of UHPC to chloride penetration and possible corrosion damage [28].

These results confirm that the use of an appropriate curing process and a reduction in the amount of water in the mixture significantly improve the resistance of UHPCs to chloride penetration. However, the variety of test conditions makes a direct comparison of the results difficult, which requires further analysis.

#### 5.1.3. Carbonation Resistance

Concrete carbonation is a chemical process in which atmospheric CO_2_ carbon dioxide reacts with cement hydration products, such as calcium hydroxide Ca(OH)_2_. This reaction produces calcium carbonate CaCO_3_, which lowers the pH of the concrete to around 9. This decrease in alkalinity results in the destruction of the protective oxide layer on the surface of reinforced steel, making it susceptible to corrosion, which, consequently, can lead to cracking of concrete structures [28,220].

However, ultra-high-performance concretes exhibit significant resistance to carbonation, mainly due to their exceptionally dense structure and low water-to-binder ratio, which limit the penetration of CO_2_ into the material. Studies conducted by Long et al. [73] showed that under sealed, standard, and heat-curing conditions, carbonation does not occur in UHPC. Another study found that the average depth of carbonation in UHPC after 28 days was less than 0.30 mm [221], which confirms its high resistance to this process.

### 5.2. Resistance to Freeze–Thaw Cycles

Mehta [222] indicated that the main factors that cause concrete degradation are reinforcement corrosion, cyclic freezing and thawing, and physicochemical interactions in the erosive environment. Therefore, the resistance to frost and resistance to corrosion of hybrid fiber-reinforced concrete are key aspects of its durability.

Research conducted by Guler et al. [74] showed that different types of fibers effectively inhibit the initiation and propagation of cracks in the concrete matrix due to the effect of the bridge. Fibers also strengthen the connection between the matrix and the internal reinforcement, leading to greater resistance of the concrete to freeze–thaw cycles [75]. Similar conclusions were formulated by Yao et al. [223], who found that the use of hybrid fibers of FST-PV reduces the number of fine pores in the concrete structure, improves its cohesion, and increases resistance to abrasion. In addition, increasing tensile strength and limiting microcrack propagation translate into improved frost resistance and corrosion resistance. The pore structure of concrete has a significant impact on its durability. Some researchers also emphasize that the way hybrid fibers are mixed affects the frost resistance of concrete. Li et al. [224] conducted research showing that the introduction of hybrid fibers into the layered structure of concrete allows a three-dimensional reinforcement effect within the layer interfaces. As a result, the number of discontinuities at the interface of individual layers is reduced, improving frost resistance compared to plain concrete. Although there are numerous studies on the effect of pore structure on frost resistance, quantitative analysis of the relationship between pore structure and corrosion resistance is still limited, indicating the need for further research in this area.

In the context of UHPC, the main cause of its degradation in cold climates is the freeze–thaw cycles. Due to the exceptional structural density of UHPC, external water cannot easily penetrate the interior of the material, significantly reducing the risk of damage. The introduction of steel fibers also inhibits the development of microcracks [225]. The test results showed that after 600 freeze–thaw cycles, the durability factor of UHPC remained ≥ 100, and the weight loss was minimal [222]. Furthermore, Smarzewski and Barnat-Hunek [226] found that the hybridization of steel and polypropylene fibers increases the resistance of UHPC to freeze–thaw cycles compared to concrete containing a single type of fiber at the same volume content. The results of the above research confirm the key role of the pore structure and fiber composition in shaping the resistance to frost and corrosion of modern high-durability concretes.

Although laboratory studies consistently demonstrate the high resistance of hybrid fiber-reinforced UHPC to chloride intrusion, carbonation, and freeze–thaw cycles, a comprehensive assessment of its long-term durability also requires field observations under real environmental conditions. In marine environments, where structures are exposed to high concentrations of chlorides, fluctuating humidity, and dynamic mechanical loads, UHPC shows great potential in mitigating corrosion processes due to its extremely low permeability and its ability to control crack development. Similarly, in temperate and cold climates, where cyclic freezing and thawing in the presence of de-icing salts poses a major threat, hybrid reinforcement with steel and synthetic fibers helps maintain the structural integrity of the material by preventing frost-induced damage and limiting mass loss.

However, despite the promising results of accelerated laboratory tests, real-world exposure conditions often involve significantly more complex degradation mechanisms, including long-term temperature variations, aggressive gases, dynamic loading, and mechanical damage. Therefore, long-term studies are necessary to better understand the interaction between concrete microstructure, fiber reinforcement type, and actual environmental exposure. Such analyses will enable a more accurate evaluation of UHPC performance in structures subjected to multifactorial degradation and will support further optimization of its composition for specific applications.

### 5.3. Fire Resistance

Concrete is an excellent fire-resistant material, mainly due to its low thermal conductivity and high heat capacity. However, studies show that high- and ultra-high-performance concrete samples begin to crack explosively at temperatures above 600 °C [227]. When heated to 200–300 °C, the compressive strength of UHPC initially increased but then began to decrease [76]. After testing at 500 ± 50 °C for 60 min, the compressive strength of the three types of concrete decreased significantly, reaching 62.2% for UHPC, 46.7% for HPC, and 58.5% for OC. After 120 min of exposure at the same temperature, the residual compressive strength was 55.6%, 34.6%, and 52.7% of the initial strength, respectively. Furthermore, the study shows that the internal temperatures of the UHPC samples were always higher than those of HPC and OC, suggesting smaller temperature gradients between the core and periphery of the UHPC concrete, which could contribute to reducing internal thermal stresses. Furthermore, when exposed to the same fire temperature and duration, UHPC showed a lower mass loss compared to HPC and OC.

The steel fiber content in UHPC concrete can help reduce strength loss by inhibiting crack propagation. Studies by Tai et al. [76] showed that the compressive strength of UHPC with 1%, 2%, and 3% steel fibers improved by 24%, 4%, and 7%, respectively, after heating to 200 °C. After heating to 800 °C, the compressive strength decreased by approximately 82%, 78%, and 77%, respectively, which was approximately 20% of the initial strength. Zheng et al. [77] found that after heating to 100 °C, the compressive strength of UHPC with 1%, 2%, and 3% steel fibers decreased by 28.1%, 26.1%, and 18.8%, respectively. After heating to 600 °C, the compressive strength of the UHPC samples with added steel fibers was approximately 37.9%, 43%, and 33.5% of the initial strength, respectively. After heating to 800 °C, the UHPC underwent severe deformation, leading to a loss of performance. However, the mass loss after heating UHPC samples with different steel fiber content was less than 12% of the initial mass [228]. By appropriately selecting low melting point fibers, explosive spalling can be reduced, which is the result of the formation of pores and channels by synthetic fibers that melt at a temperature of around 150 °C, allowing the release of steam and minimizing the pressure increase inside the pores [229]. The fire resistance mechanism of polypropylene fibers in UHPC is primarily related to this behavior. When exposed to temperatures above their melting point (~150–170 °C), polypropylene fibers melt and disappear from the concrete matrix, leaving behind a network of fine channels and pores. These voids act as escape routes for water vapor generated during heating, reducing internal pore pressure and thus mitigating the risk of explosive spalling. This mechanism is particularly important in UHPC, which is characterized by a dense, low-permeability matrix that otherwise restricts moisture migration. Without such pathways, thermal stress and vapor build-up can cause sudden and violent cracking. Although the inclusion of polypropylene fibers may slightly reduce compressive strength at lower temperatures, their presence significantly enhances fire performance by relieving internal pressure and reducing structural damage. Microstructural studies confirm that UHPC with polypropylene fibers exhibits increased permeability and improved structural connectivity due to this pore formation [230]. The synergistic use of steel and polypropylene fibers further enhances performance by combining crack-bridging capacity with vapor pressure mitigation.

The study by Zheng et al. [231] indicates that the use of polypropylene fibers in UHPC has a negative effect on compressive strength at lower temperatures but improves it at higher temperatures. Heinz et al. [78] evaluated the fire resistance of UHPC with the addition of steel and polypropylene fibers, finding that the best results were obtained for samples containing 3.05% steel fibers and 0.6% polypropylene fibers. Peng et al. [80] showed that the addition of 0.9% polypropylene fibers significantly improved the fire resistance of UHPC. The combination of steel and polypropylene fibers reduced or eliminated spalling during fire exposure and significantly reduced loss of strength due to cracking [232,233]. In turn, microstructural studies have shown that UHPC with steel and polypropylene fibers exhibits higher permeability and better structural connectivity due to the formation of pores and channels associated with the melting of polypropylene fibers [230].

The use of low-thermal-expansion aggregates can help prevent spalling caused by thermal stresses [234]. However, the introduction of additional water by using conditioned aggregates with a high moisture content, e.g., saturated lightweight aggregates, can lead to severe spalling [28]. The studies carried out indicate a significant impact of various additives, such as steel and polypropylene fibers, on improving the fire resistance of UHPC. Further research can focus on optimizing the composition of concrete to achieve even better results in terms of strength and resistance to high temperatures while minimizing the risk of explosive spalling.

Additionally, in the context of UHPC fire resistance, it is relevant to compare its performance with other fire-resistant materials and protective systems. Although refractory bricks offer excellent thermal resistance at temperatures above 1000 °C, they lack the mechanical strength required for structural applications. Traditional concrete, although less strong than UHPC, often exhibits reduced spalling due to higher porosity. Similarly, geopolymer concretes are gaining interest as a viable alternative because of their superior thermal stability and lower risk of explosive spalling. Moreover, passive fire protection systems, such as intumescent coatings, fireproof mortars, or ceramic claddings, are commonly used to delay heat transfer in structural elements. Although not routinely applied to UHPC, their integration could further enhance their fire resistance. Recent research suggests that combining steel and polypropylene fibers with such external protective systems can provide a hybrid solution, mitigating both internal thermal stress and surface damage during exposure to fires. In light of this, future work should not only address optimization of the composition of UHPCs but also investigate the synergetic potential of combining UHPC with passive fire protection technologies to ensure comprehensive structural safety under high-temperature conditions.

## 6. Costs and Environmental Impact of Using Fibers in UHPC

### 6.1. Economic Aspects of Using Hybrid Fibers in UHPC

Despite excellent mechanical and durability properties, the use of fibers in UHPC is associated with significant economic challenges. In the early stages of UHPC development, the cost issue was considered secondary to achieving material reliability in engineering applications. As studies indicate, the higher material costs of UHPC can potentially be compensated by reduced concrete consumption, reduced reinforcement, simplified formwork, and reduced transport and labor costs due to thinner cross-sections and improved workability [28,235]. In practice, however, the largest share in the unit costs of UHPC is taken by a high dose of Portland cement, mineral additives such as silica fume, and, importantly, in the context of this work—the addition of fibers. High-strength steel fibers constitute a particularly significant percentage of total mix costs, often exceeding the costs of cement itself. For this reason, fibers can be a limiting factor in the implementation of UHPC in the public sector, especially in developing countries where project budgets are limited [236]. In addition to the material costs, logistical and technological costs cannot be ignored. The production of UHPC with the addition of steel fibers requires a concrete plant with a special infrastructure, including additional fiber hoppers and high-performance mixers to evenly distribute the fibers in the mix. This means additional capital investment is necessary to implement production on a larger scale [28].

In response to these limitations, the literature suggests a number of strategies to reduce the cost of UHPC containing fibers.

1. Reducing the dosage of steel fibers without compromising mechanical properties by combining them with cheaper, locally available synthetic or natural fibers.
2. Incorporating coarse aggregates to reduce the share of the expensive powder part of the mix.
3. Eliminating heat treatment and other energy-intensive processes, such as high-pressure compaction.
4. Designing mixes based on locally available materials, for example, fly ash, metakaolin, or lime powder, that can replace up to 50% of Portland cement without significant loss of mechanical properties.

The use of optimized fiber-based blends can significantly reduce the total cost of UHPC—estimated from USD 1500–3000/m^3^ (for proprietary, commercial blends) up to USD 600–850/m^3^ for non-proprietary blends, including fiber-reinforced blends [237]. A study by Arora et al. [238] showed that the cost of UHPC can be significantly reduced by using a compressible filler model and by designing the binder based on microstructural and rheological criteria.

Furthermore, the use of fiber combinations can provide long-term savings by improving durability and reducing maintenance costs. For example, steel fibers improve the fracture toughness of UHPC, while synthetic fibers reduce its brittleness, which, together, contribute to extending the service life of the structure. It is worth noting that a similar approach to estimating costs early in material development was used in the analysis of fly ash-based adsorbents, where “a cost estimation approach was necessary to identify the most cost-effective design strategies—before production facilities are built” [239]. Similarly, in the context of fiber-based UHPC, early analysis of material costs, feedstock availability, and required infrastructure is crucial for the sustainable implementation of this technology at an industrial scale. A similar approach, based on early cost estimation and assessment of implementation feasibility, has also been used in other sectors of materials technology. As the authors of [240] noted in their paper, "comparing the costs of activated carbon synthesis using industrial waste allows the identification of cost-effective options before pilot or industrial investments". Such analyses are crucial in the context of modern engineering materials and are a starting point for their adaptation in practice, which has a direct impact on the realities of implementing UHPC with the addition of hybrid fibers.

### 6.2. Environmental Aspects of Using Fibers in UHPC

The growing awareness of environmental issues and the scale of emissions related to the construction industry force the implementation of solutions that support sustainable development. In 2020, the construction sector was responsible for 11.7 gigatons of CO_2_ emissions, which constituted up to 37% of global greenhouse gas emissions, and concrete production reached 14 billion m^3^ [241]. The main source of emissions is cement, a component of concrete that accounts for up to 85–90% of its carbon footprint [242]. One of the methods to reduce the negative impact of concrete production on the environment is the addition of fibers to the cement mix, which leads to the creation of fiber-reinforced concrete. Fibers not only improve the mechanical properties of concrete (especially its tensile and bending strength) but also allow for a reduction in the cement content in the mix without compromising strength properties, which leads to a reduction in CO_2_ emissions [243]. Importantly, the use of fibers as a component of UHPC concrete is not limited to conventional materials. Recycled fibers, such as steel fibers from tires, elements of shredded wind turbine blades, or natural coconut and bamboo fibers, are increasingly being used [243,244,245,246,247,248,249,250,251]. This reduces the demand for primary raw materials and reduces the amount of waste that goes to landfills.

Introducing fibers into concrete can also be combined with other pro-ecological solutions, such as using waste materials instead of cement and aggregates. Both strategies can be implemented in parallel, which also increases the environmental efficiency [252,253]. However, it should be noted that the impact of these actions is not always clear-cut: the use recycled fibers does not necessarily lead to a reduction in the carbon footprint if it is not accompanied by a reduction in cement [254].

To comprehensively assess the environmental impact of different types of fibers and the way they are used in UHPC, a life cycle analysis (LCA) is necessary. This method allows for an objective comparison of solutions throughout the entire life cycle of the material—from the extraction of raw material, through production and use, to disposal (EoL) [125,128,255]. It makes it possible to identify the most environmentally beneficial strategies for the use of FRC.

## 7. Summary and Conclusions

### 7.1. Conclusions of the Review

This article reviews the literature on the influence of hybrid fibers on the mechanical and durability properties of UHPCs, taking into account experimental studies and new directions of development in this field. The selection of materials was based on the analysis of the parameters of the fiber and the experimental methods, which allowed for the creation of a comprehensive picture of the current state of knowledge. The conclusions of this review are as follows.

(1) The use of hybrid steel fibers in ultra-high-performance concrete (UHPC) significantly improves its mechanical properties, especially compressive, tensile, and bending strength, due to the synergy of short fibers that bridge microcracks and long fibers that limit the development of macrocracks. The appropriate selection of the proportions and types of fibers allows for an increase in the modulus of elasticity and an improvement in the ductility and ability of the concrete to crack multiple times, making it more resistant to dynamic loads and long-term use.
(2) Hybrid steel–synthetic fibers effectively improve the mechanical properties of concrete, combining the high strength of steel fibers with the flexibility and ability to bridge microcracks of synthetic fibers. The use of such combinations increases the tensile strength, fracture energy, and ductility of concrete while reducing the brittleness of the material. However, an excessive amount of synthetic fibers can lead to a decrease in compressive strength and the modulus of elasticity, which is why it is crucial to properly select the proportions of both types of fibers in the mixture.
(3) Short basalt–polypropylene hybrid fibers, despite reducing the compressive strength of high-performance concrete, significantly improve its tensile strength and crack resistance, indicating their beneficial effect in applications requiring increased durability and resistance to microcracks.
(4) Hybrid fibers can improve or worsen the permeability of concrete depending on their quantity and environmental conditions—in optimal proportions (0.05–0.1%), they reduce microcracks and improve water tightness, but their excess (>0.2%) increases porosity and can weaken the structure, especially at high temperatures, where they promote the formation of microchannels facilitating water flow.
(5) The use of hybrid fibers in UHPC can also increase its resistance to chloride ion penetration by reducing porosity and limiting the formation of microcracks, leading to a lower chloride diffusion coefficient and increased durability of concrete structures.
(6) Hybrid fibers, by increasing the structural density of concrete and reducing microcracks, can also improve the resistance of concrete to carbonation, limiting CO_2_ penetration and thus minimizing the impact of this process on the durability of the structure.
(7) The introduction of hybrid fibers into concrete effectively increases its resistance to freeze–thaw cycles by reducing cracks due to the bridging effect, improving the cohesion of the structure and optimizing the pore system, which limits the negative impact of frost and increases the durability of the material.
(8) The use of hybrid fibers, which combine steel and polypropylene fibers, significantly improves the fire resistance of UHPC, reducing the risk of spalling and loss of strength by reducing thermal stresses and creating pores that facilitate the release of water vapor.

### 7.2. Future Research Directions

In light of the growing demand for sustainable construction materials, future research on UHPC should emphasize the integration of environmentally friendly supplementary cementitious materials (SCMs) with hybrid fiber reinforcement systems. Such combinations have the potential to reduce the carbon footprint of the UHPC while simultaneously enhancing its mechanical performance and durability, particularly in aggressive environments. Understanding the synergy between green additives and hybrid fiber systems is essential for optimizing crack resistance, toughness, and long-term structural integrity.

It is recommended to conduct further research on the optimal ratio and arrangement of hybrid steel fibers of different lengths, diameters, and shapes in UHPC to maximize its mechanical strength. In particular, it is necessary to investigate how the interaction of short and long fibers affects the mechanism of microcrack bridging and macrocrack development and how different types and proportions of fibers affect the modulus of elasticity and resistance of concrete to degradation under long-term loading.

Another important research direction is the crack-bridging and fiber-pulling mechanisms in concrete containing steel and synthetic fibers. Special attention should be paid to the influence of the ratio of steel and synthetic fibers on the compressive strength, tensile strength, and fracture energy of concrete to determine the optimal combinations that increase both the strength and the ductility of UHPC concrete.

In addition, research is recommended on the optimization of the composition of high-performance concrete mixtures with basalt–synthetic hybrid fibers. Particular attention should be paid to the influence of different proportions of these fibers on the compaction process and the microstructure of concrete. These studies should also include modification of the parameters of the mixture, such as the use of plasticizing admixtures or new compaction methods, to minimize the negative influence of fibers on compressive strength while maintaining the favorable properties of concrete in terms of tensile strength and fracture energy.

Further research on UHPCs should also focus on their long-term durability in real environmental conditions, taking into account the influence of frost, chlorides, and aggressive chemicals. An important area of research is also the modeling of moisture and ion transport processes in UHPC, which will allow for better prediction of its behavior over time. The composition of the mixture should also be optimized, looking for ecological additives, and the influence of different types of fibers on the microstructure of the concrete should be analyzed. It is also worth developing self-healing technologies that can significantly increase the durability of UHPC.

Such research directions align not only with the principles of sustainable development but also with the advancement of next-generation UHPC composites geared to sustainability, resilience, and environmental responsibility. These investigations will provide critical information on material design strategies that address both performance and ecological impact.

### 7.3. Practical Recommendations for Engineering Applications

Based on the analysis of the literature and experimental results, recommendations for the selection of hybrid fibers for UHPC were formulated based on practical applications. For elements exposed to dynamic loads, such as bridges or prefabricated infrastructure elements, it is recommended to use a combination of short (6–13 mm) and long (30–50 mm) steel fibers in a ratio of 1:1 or 2:1 or in the amount of 1.5% to 2.0% by volume. Such a selection improves the mechanical properties of concrete, supporting the bridging of microcracks and limiting the development of macrocracks.

In structures used in an aggressive chemical environment, such as tunnels or bridge surfaces, it is recommended to use hybrid steel–synthetic fibers in a ratio of 3:1 or in an amount of 1.0–1.5% by volume. This composition improves the chemical resistance of the concrete and its rigidity. For structures requiring fire resistance, such as tunnel elements, steel fibers (1%) and polypropylene fibers (0.1–0.2%) are used. Polypropylene reduces thermal stress, reducing the risk of thermal explosion.

In highly durable structures, such as tanks or hydrotechnical structures, it is recommended to use short basalt or synthetic fibers in the amount of 0.1% to 0.3% to improve resistance to microcracks and reduce the porosity of the concrete. For precast concrete elements, where good workability of the mix and surface quality are important, short steel fibers (13 mm) in the amount of 1.5% to 2.0% by volume will be the best, ensuring the appropriate rheological properties of concrete.

The selection of appropriate fibers for UHPC concrete depends on the specific requirements of the structure, which allows for the optimization of its properties in various operating conditions.

## Figures and Tables

**Table 1:** Summary of key information related to the mechanical studies reviewed.

Author	Types of Fibers	Total Volume Fractions [%]	Test Methods
Park et al. (2012) [56]	steel–steel	1.5 ÷ 2.5	tensile strength
Kang et al. (2016) [57]	steel–synthetic	1.5	compressive strength, tensile strength
Yoo et al. (2017) [51]	steel–steel	2	compressive strength
Smarzewski and Barnat-Hunek (2018) [58]	steel–synthetic	1	compressive strength, tensile strength, flexural strength, modulus of elasticity, energy cracking
Smarzewski (2019) [59]	basalt–synthetic	1 ÷ 2	compressive strength, tensile strength, flexural strength, energy cracking
Türker et al. (2019) [60]	steel–steel	1.5	compressive strength, flexural strength, modulus of elasticity
Wang et al. (2019) [61]	basalt–synthetic	0.125 ÷ 0.242	compressive strength, tensile strength, flexural strength, modulus of elasticity
Deng et al. (2021) [62]	basalt–synthetic	0.3 ÷ 0.55	flexural strength
Aisheh et al. (2022) [41]	steel–synthetic	1.25 + 2.25	compressive strength, tensile strength, flexural strength, modulus of elasticity
Akça and İpek (2022) [63]	steel–steel	3	compressive strength, tensile strength, modulus of elasticity
Elsayed et al. (2022) [64]	steel–synthetic	-	compressive strength, tensile strength, flexural strength
Yu et al. (2022) [65]	steel–steel	2	compressive strength, flexural strength
Bian et al. (2024) [66]	steel–synthetic	1.5	compressive strength, tensile strength, flexural strength, modulus of elasticity, energy cracking

**Table 2:** Summary of the most important information regarding the durability of UHPC.

Author	Research Area	Type of Research
Li et al. [67] Xu et al. [68] Yang et al. [69] Liu [70]	permeability	permeability tests with hybrid fibers
Hung [71]		permeability test at elevated temperature
Ghafari et al. [72]	resistance to chloride and salt penetration	corrosion test
Long et al. [73]	carbonation resistance	CO_2_ penetration
Guler et al. [74] Yao et al. [75] Smarzewski and Barnat-Hunek [58]	resistance to freeze–thaw cycles	freeze–thaw cycle test
Tai et al. [76] Zheng et al. [77] Heinz et al. [78] Peng et al. [79]	fire resistance	high-temperature resistance test

**Table 3:** Summary of test results for steel–steel hybrid fibers.

Ref.	Fiber Types	Fibers Volume Fraction [%]	l_n_/d_n_ [mm]	f_c_ [MPa]	f_t_ [MPa]	f_l_ [MPa]	E_c_ [GPa]
[63]	SSF + LHF	2 + 1	6/0.20; 35/0.75	158.11	23.73		62.5
	SSF + LHF	2 + 1	6/0.20; 35/0.75	130.41	21.12		
[65]	LSF + SSF	1.5 + 0.5	13/20; 6/0.16	142		30.9	
	SSF + LHF	0.5 + 1.5	6/0.16; 35/0.55	136.5			
	LSF + SSF	1 + 1	13/20; 6/0.16	135		27	
	LSF + SSF + LHF	0.125 + 0.375 + 1.5	13/20; 6/0.16; 35/0.55	132		23	
	LSF + LHF	0.5 + 1.5	13/20; 35/0.55	130			
	LSF + SSF	0.5 + 1.5	13/20; 6/0.16	128		25.50	
[60]	SSF + LHF	1.0 + 0.5	13/0.16; 60/0.75	164		11.18	43
[51]	LSF + LTF	1.5 + 0.5	19.5/0.2; 30/0.30	202.2			
	LSF + LHF	1.5 + 0.5	19.5/0.2; 30/0.38	199			
	LSF + LTF	1.0 + 1.0	19.5/0.2; 30/0.30	198.9			
	LSF + LHF	1.0 + 1.0	19.5/0.2; 30/0.38	187.9			
	LSF + LHF	0.5 + 1.5	19.5/0.2; 30/0.38	186.1			
	LSF + LTF	0.5 + 1.5	19.5/0.2; 30/0.30	185.4			
[56]	LTF + SSF	1.0 + 1.5	30/0.3; 13/0.2		18.56		
	LTF + SSF	1.0 + 1.0	30/0.3; 13/0.2		14.77		
	SHF + SSF	1.0 + 1.5	30/0.375; 13/0.2		13.84		
	LTF + SSF	1.0 + 0.5	30/0.3; 13/0.2		13.50		
	LSF + SSF	1.0 + 1.0	30/0.3; 13/0.2		13.31		
	LSF + SSF	1.0 + 1.5	30/0.3; 13/0.2		13.22		
	SHF + SSF	1.0 + 1.0	30/0.375; 13/0.2		12.25		
	LHF + SSF	1.0 + 1.5	62/0.775; 13/0.2		12.01		
	LSF + SSF	1.0 + 0.5	30/0.3; 13/0.2		11.42		
	LHF + SSF	1.0 + 1.0	62/0.775; 13/0.2		11.33		
	SHF + SSF	1.0 + 0.5	30/0.375; 13/0.2		10.90		
	LHF + SSF	1.0 + 0.5	62/0.775; 13/0.2		10.31		

Note: SSFs—short straight fibers; LHFs—long hooked fibers; LSFs—long straight fibers; LTFs—long twisted gibers; SHFs—short hooked fibers; l_n_—fiber length; d_n_—fiber diameter; f_c_—compressive strength; f_t_—tensile strength; f_l_—flexural strength; E_c_—modulus of elasticity.

**Table 4:** Summary of test results for steel–synthetic hybrid fibers.

Ref.	Fiber Types	Fiber Volume Fraction [%]	l_n_/d_n_ [mm]	f_c_ [MPa]	f_t_ [MPa]	f_l_ [MPa]	E_c_ [GPa]	G_f_ [N/mm]
[66]	StSF + PeSF	1.0 + 0.5	13/0.2; 18/0.025	104	8.87	12.69	33.7	4
	StSF + BSF	1.0 + 0.5	13/0.2; 18/0.024	103.7	6.60	9.25	33.9	0.77
	StSF + BSF + PeSF	0.5 + 0.5 + 0.5	13/0.2; 18/0.024; 18/0.025	103	6.83	10.65	32.6	3.77
	StSF + BSF	0.5 + 1.0	13/0.2; 18/0.024	92.8	4.92	4.77	32.7	3.26
	StSF + PeSF	0.5 + 1.0	13/0.2; 18/0.025	89.8	8.50	13.57	32.8	6.38
[58]	StHF + PSF	0.75 + 0.25	50/1; 12/0.025	144.7	13.50	9.80	34.27	34.26
	StHF + PSF	0.5 + 0.5	50/1; 12/0.025	133.9	10	9.20	32.45	32.29
	StHF + PSF	0.25 + 0.75	50/1; 12/0.025	122.3	9.30	8.60	29.63	29.93
[41]	StSF + PSF	2.0 + 0.25	15/0.12; 8/0.033	175	7.70	13.70	48	
	StSF + PSF	1.0 + 0.25	15/0.12; 8/0.033	159	7.50	11.70	47	
	StSF + PSF	1.5 + 0.25	15/0.12; 8/0.033	158	8.50	12.80	48	
[64]	StHF + PSF		50/0.1; 12/0.018	64.2	7.10	15.20		
	StHF + GSF		50/0.1; 12/0.013	60.3	7.60	14.90		
	GSF + PSF		12/0.013; 12/0.018	56.9	5.20	14.10		
	StHF + PSF		50/0.1; 12/0.018	44.7	5.10	9.80		
	StHF + GSF		50/0.1; 12/0.013	40.3	5.70	10.60		
	GSF + PSF		12/0.013; 12/0.018	39.3	2.70	6.90		
[57]	SLSF + SSSF	1.0 + 0.5	19.5/0.2; 16.3/0.2	149	14.30			
	SLSF + SSSF + PvaSF	0.67 + 0.33 + 0.5	19.5/0.2; 16.3/0.2; 12/0.040	143	11.84			
	SLSF + SSSF + PeSF	0.67 + 0.33 + 0.5	19.5/0.2; 16.3/0.2; 18/0.012	142	16.21			
	SLSF + SSSF + BSF	0.67 + 0.33 + 0.5	19.5/0.2; 16.3/0.2; 12/0.012	128	14.74			

Note: StSFs—steel straight fibers; BSFs—basalt straight fibers; PeSFs—polyethylene straight fibers; PSFs—polypropylene straight fibers; StHFs—steel hooked fibers; GSFs—glass straight fibers; SLSFs—steel long straight fibers; SSSFs—steel short straight fibers; PvaSFs—polyvinyl alcohol straight fibers; G_f_—fracture energy.

**Table 5:** Summary of research results for basalt–synthetic hybrid fibers.

Ref.	Fiber Types	Fiber Volume Fraction [%]	l_n_/d_n_ [mm]	f_c_ [MPa]	f_t_ [MPa]	f_l_ [MPa]	E_c_ [GPa]	G_f_ [N/mm]
[59]	BSF + PSF	0.75 + 0.25	12/0.013; 12/0.025	114	6.8	10		17.55
	BSF + PSF	0.25 + 0.75	12/0.013; 12/0.025	116.9	8.2	9.3		17.21
	BSF + PSF	0.5 + 0.5	12/0.013; 12/0.025	115.7	8.1	9.4		17.21
	BSF + PSF	0.5 + 1.5	12/0.013; 12/0.025	111.6	8.7	9.4		15.53
	BSF + PSF	1.5 + 0.5	12/0.013; 12/0.025	107.5	7.8	11.5		14.85
	BSF + PSF	1.0 + 1.0	12/0.013; 12/0.025	106.6	8.3	9.7		12.83
[62]	BSF + PTF	0.07 + 0.42	19/0.0013; 50/0.8			5.70		
	BSF + PTF	0.04 + 0.51	19/0.0013; 50/0.8			5.20		
	BSF + PTF	0.11 + 0.32	19/0.0013; 50/0.8			5.10		
	BSF + PTF	0.17 + 0.13	19/0.0013; 50/0.8			4.90		
	BSF + PTF	0.15 + 0.21	19/0.0013; 50/0.8			4.60		
[61]	BSF + PSF	0.15 + 0.033	12/0.012; 12/0.02	110.07	6.24	10.19	40.8	
	BSF + PSF	0.15 + 0.042	12/0.012; 12/0.02	100.3	5.59	9.53	41.3	
	BSF + PSF	0.15 + 0.025	12/0.012; 12/0.02	98.13	6.39	10.47	35.5	
	BSF + PSF	0.2 + 0.025	12/0.012; 12/0.02	97.33	5.6	8.80	40	
	BSF + PSF	0.1 + 0.025	12/0.012; 12/0.02	94.67	5.19	9.74	43	
	BSF + PSF	0.2 + 0.033	12/0.012; 12/0.02	94.37	5.47	8.71	42.4	
	BSF + PSF	0.1 + 0.033	12/0.012; 12/0.02	89.57	5.33	9.90	32.9	
	BSF + PSF	0.2 + 0.042	12/0.012; 12/0.02	88.47	5.45	8.33	40.9	
	BSF + PSF	0.1 + 0.042	12/0.012; 12/0.02	87.13	5.11	10.01	41.5	

**Table 6:** Summary of test results of UHPC with hybrid fibers.

Hybrid Fibers	Ref.	Fiber Types	Fiber Volume Fraction [%]	l_n_/d_n_ [mm]	f_c_ [MPa]	f_t_ [MPa]	f_l_ [MPa]	E_c_ [GPa]
steel–steel	[64]	SSF + LHF	2 + 1	6/0.20; 35/0.75		23.73		62.5
	[66]	LSF + SSF	1.5 + 0.5	13/20; 6/0.16			30.9	
	[52]	LSF + LTF	1.5 + 0.5	19.5/0.2; 30/0.30	202.2			
steel–synthetic	[41]	StSF + PSF	2.0 + 0.25	15/0.12; 8/0.033	175			
	[58]	SLSF + SSSF + PeSF	0.67 + 0.33 + 0.5	19.5/0.2; 16.3/0.2; 18/0.012		16.21		
	[65]	StHF + PSF		50/0.1; 12/0.018			15.20	
	[41]	StSF + PSF	1.5 + 0.25	15/0.12; 8/0.033				48
basalt–synthetic	[60]	BSF + PSF	0.25 + 0.75	12/0.013; 12/0.025	116.9			
	[60]	BSF + PSF	0.5 + 1.5	12/0.013; 12/0.025		8.7		
	[62]	BSF + PSF	0.15 + 0.025	12/0.012; 12/0.02			10.47	
	[62]	BSF + PSF	0.1 + 0.025	12/0.012; 12/0.02				43

## Data Availability

The raw data supporting the conclusions of this article will be made available by the authors on request.
